# Characterization of a *C9orf72* Knockout *Danio rerio* model for ALS and cross-species validation of potential therapeutics screened in *Caenorhabditis elegans*

**DOI:** 10.1371/journal.pone.0346613

**Published:** 2026-04-10

**Authors:** Alexandre Emond, Carl Laflamme, Martine Therrien, Meijiang Liao, Claudia Maios, Audrey Labarre, Pierre Drapeau, J. Alex Parker

**Affiliations:** 1 CHUM Research Center (CRCHUM), Montreal, Quebec, Canada; 2 Department of Neuroscience, University of Montreal, Montreal, Quebec, Canada; 3 Structural Genomics Consortium, Montreal Neurological Institute-Hospital (The Neuro), McGill University, Montreal, Quebec, Canada; 4 Neurodegenerative Diseases Research Group, Department of Neurology and Neurosurgery, Montreal Neurological Institute-Hospital (The Neuro), McGill University, Montreal, Quebec, Canada; 5 Center for Neuroscience, University of California, Davis, California, United States of America; Amity University, INDIA

## Abstract

Intronic hexanucleotide repeat expansions in the *C9orf72* gene represent the most common genetic cause of the neurodegenerative diseases amyotrophic lateral sclerosis (ALS) and frontotemporal dementia. This expansion decreases *C9orf72* expression in affected patients, indicating that loss of *C9orf72* function (LOF) acts as a pathogenic mechanism. Several models using *Danio rerio* (zebrafish) for *C9orf72* depletion have been developed to explore disease mechanisms and the consequences of *C9orf72* LOF. However, inconsistencies exist in reported phenotypes, and many have yet to be validated in stable germline ablation models. To address this, we created a zebrafish *C9orf72* knockout model using CRISPR/Cas9. The *C9orf72* LOF model demonstrates, in a generally dose-dependent manner, increased larval mortality, persistent growth reduction, and motor deficits. Additionally, homozygous *C9orf72* LOF larvae exhibited mild overbranching of spinal motoneurons. To identify potential therapeutic compounds, we performed a screen on an established *Caenorhabditis elegans* (*C. elegans*) *C9orf72* homologue (*alfa-1*) LOF model, identifying 12 compounds that enhanced motility, reduced neurodegeneration, and alleviated paralysis phenotypes. Motivated by the shared motor phenotype, 2 of those compounds were tested in our zebrafish *C9orf72* LOF model. Pizotifen malate was found to significantly improve motor deficits in *C9orf72* LOF zebrafish larvae. We introduce a novel zebrafish *C9orf72* knockout model that exhibits phenotypic differences from depletion models, providing a valuable tool for *in vivo C9orf72* research and ALS therapeutic validation. Furthermore, we identify pizotifen malate as a promising compound for further preclinical evaluation.

## Introduction

Amyotrophic lateral sclerosis (ALS) is a devastating neuromuscular disorder characterized by progressive degeneration of motor neurons (MNs). Approved treatments provide only limited benefits, and death typically occurs within 2–3 years of symptom onset [1]. A GGGGCC hexanucleotide repeat expansion in the first intron of *C9orf72* is the most common genetic cause of ALS (*C9*-ALS) and frontotemporal dementia [2–4]. This expansion is proposed to contribute to ALS pathogenesis through three non-mutually exclusive mechanisms: (1) RNA toxicity via sense and antisense foci, (2) protein toxicity through dipeptide repeat proteins generated by repeat-associated non-ATG translation, and (3) *C9orf72* loss-of-function (LOF), as evidenced by reduced mRNA and protein levels in patient-derived tissues [2,5,6]. Mammalian gain-of-function (GOF) models often fail to fully replicate C9orf72-ALS histopathological and behavioral phenotypes [7]. LOF remains comparatively understudied, and the full biological function of C9orf72 is still unclear [5].

LOF animal models have provided critical insights into *C9orf72* function and ALS pathogenesis. However, *C9orf72* knockout (KO) mouse models fail to fully recapitulate *C9*-ALS pathology, lacking MN loss, exhibiting broad immune dysregulation, and presenting inconsistent motor phenotypes [8–11]. Whether mice have intrinsic resilience to *C9orf72* LOF-induced motor deficits and MN degeneration remains unclear. This highlights the need for alternative vertebrate systems to investigate *C9orf72* LOF and model ALS pathogenesis.

Zebrafish (*Danio rerio*) are a well-established vertebrate model for neurological diseases, sharing key neuroanatomical, neurochemical, and genetic similarities with humans [12]. Many zebrafish models exhibit neurodegenerative markers, including neuronal loss and gliosis, even at early larval stages [13–16]. Zebrafish possess a single *C9orf72* ortholog with 85% sequence identity to the human protein. The first *C9orf72* knockdown (KD) model, generated using morpholinos, revealed spinal MNs axonal overbranching, shortening, and motor deficits [17]. A subsequent microRNA-based KD model demonstrated early mortality, morphological defects, synaptic dysfunction at the neuromuscular junction (NMJ), motor deficits, MNs degeneration, and muscle atrophy [18]. However, a recent CRISPR/Cas9 *C9orf72* KO model exhibited retinal disruption, neuroinflammation, and neurodegeneration in aged mutants, but no spinal MN loss or NMJ defects [19]. Validation of *C9orf72* KD models ALS-related phenotypes in stable germline KO lines is crucial to define zebrafish potential as an alternative vertebrate system for the study of *C9orf72* LOF and ALS pathogenesis.

Non-vertebrate animal models offer advantages for high-throughput compound screening, with promising hits able to be subsequently validated in higher-order systems [20]. *C. elegans* is a well-established model for neurological diseases, with a simple, well-characterized nervous system that shares conserved neuronal subtypes and molecular pathways with humans [21,22]. The sole *C. elegans* ortholog of *C9orf72*, *alfa-1*, shares 59% sequence similarity with its human counterpart. The *alfa-1* KO *C. elegans* mutant line *alfa-1*(ok3062) exhibits age-dependent paralysis and GABAergic MNs degeneration [23]. A robust motor phenotype would enable high-throughput phenotypic screening for *C9*-ALS therapeutics in this line [24–27]. Accordingly, we investigated selected early developmental and larval consequences of *C9orf72* LOF in zebrafish and used these phenotypes for cross-species validation of candidate therapeutic compounds.

In this study, we created a stable *C9orf72* KO zebrafish model using CRISPR/Cas9 to investigate ALS-related phenotypes, resolve discrepancies with existing KD models, and validate potential *C9*-ALS therapeutics. Homozygous *C9orf72*^*-/-*^ larvae displayed reduced survival, persistent growth deficits, motor impairments, and mild disruption of spinal MN axonal branching and neuromuscular junction (NMJ) postsynaptic organization. In contrast, the heterozygous *C9orf72*^*-/+*^ specimens showed milder mortality, smaller size, and motor deficits. Phenotypes for both genotypes were less severe than those reported for the KD models. Due to the zebrafish#39;s lesser suitability for high-throughput drug screening, *C. elegans* was used to identify potential *C9*-ALS therapeutics, with the best candidates subsequently validated in our *C9orf72* LOF model. We demonstrated that the *alfa-1(ok3062)* mutants exhibit rapid-onset motor deficits in liquid culture, allowing for the identification of 80 compounds that significantly improved swimming activity in young adult mutant worms. Twelve of these compounds further alleviated age-dependent paralysis and neurodegeneration in *alfa-1(ok3062)* mutants on solid media. By utilizing the motor phenotype of our *C9orf72* KO zebrafish model, we validated pizotifen malate’s (PM) effectiveness in mitigating *C9orf72* LOF-associated motor deficits. Our findings indicate that *C9orf72* LOF induces ALS-related phenotypes in zebrafish larvae, with dose-dependent severity for most phenotypes. However, we did not observe clear signs of spinal MN degeneration at the larval stage. These observations support the notion that stable genetic mutants may show significant differences in phenotypic manifestation and severity compared to RNA interference (RNAi)-based KD models.

## Results

### Generation and validation of a *C9orf72* zebrafish knockout model

**Fig 1:**
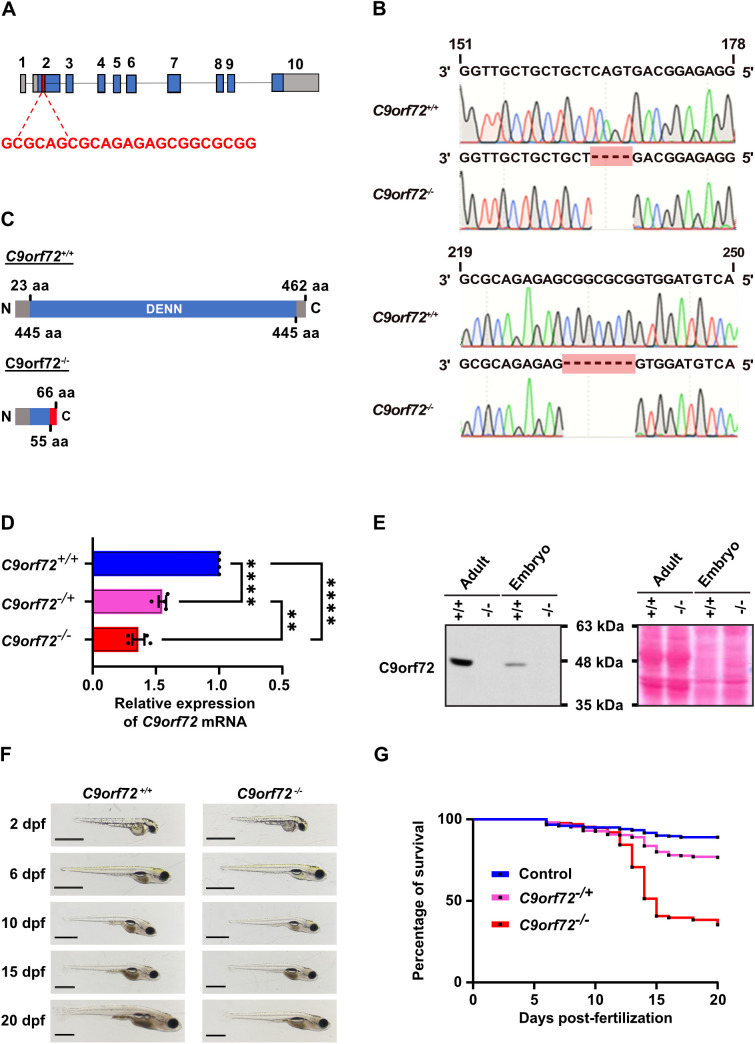
Generation and validation of a stable zebrafish *C9orf72* knockout line. **(A)** Schematic representation of the CRISPR/Cas9-mediated knockout of the endogenous *C9orf72* gene. A guide RNA was designed to target the start codon region in the second exon of *C9orf72*, inducing indels. **(B)** Sequencing of the *C9orf72* knockout line revealed two distinct deletions (Δ4 and Δ7) at different sites within the *C9orf72* sequence, resulting in a total deletion of 11 base pairs that was absent in *C9orf72*^+/+^ wild-type specimens. **(C)** Schematic representation of the predicted truncated C9orf72 protein resulting from the frameshift caused by the Δ4 and Δ7 deletions. Amino acids (aa). **(D)** Quantitative analysis of *C9orf72* mRNA expression at 2 days post-fertilization (dpf) demonstrated significantly reduced transcript levels in *C9orf72*^-/+^ and *C9orf72*^*-/-*^ compared to *C9orf72*^+/+^ controls, with *C9orf72*^*-/-*^ showing significantly lower levels than *C9orf72*^-/+^. Data are presented as mean ± SEM, normalized to *Polr2d* mRNA levels. Ordinary one-way ANOVA with Holm-Šídák#39;s multiple comparisons post-hoc test; **** *p < 0.0001*, ** p ≤ 0.01 N = 4 experimental repeats, n = 3 technical replicates. **(E)** Immunoblotting and Ponceau staining confirmed the absence of C9orf72 protein in *C9orf72*^*-/-*^ *(*-/-) adults and 2 dpf embryos. *C9orf72*^+/+^ (+/+) specimens served as controls for the presence of the protein. **(F)** Comparative morphological analysis revealed no apparent defects in *C9orf72*^*-/-*^ compared to *C9orf72*^+/+^ controls. **(G)** Kaplan–Meier survival analysis up to 20 dpf showed significantly reduced survival in *C9orf72*^-/+^ and *C9orf72*^*-/-*^ compared to *C9orf72*^+/+^ controls after 10 dpf, with *C9orf72*^*-/-*^ exhibiting significantly lower survival than *C9orf72*^-/+^ after 13 dpf. Log-rank (Mantel-Cox) test; N = 3 experimental repeats, n = 300 total fish per genotype. Scale bars = 1 mm.

Zebrafish have a single conserved ortholog of *C9orf72*, located on chromosome 13 [18]. Using CRISPR/Cas9-mediated genome editing, we generated a *C9orf72* KO line harboring two distinct deletions (Δ4 and Δ7) within the second exon, resulting in a combined deletion of 11 base pairs compared to wild-type (*C9orf72*^*+/+*^) specimens (Fig 1A**-B**). These deletions are predicted to cause a frameshift mutation, resulting in premature translation termination and a truncated protein lacking more than 85% of the zebrafish C9orf72 sequence (**Fig 1C**). To minimize off-target effects, heterozygous *C9orf72*^*-/+*^ F1 individuals were outcrossed with wild-type specimens, and F2 incrossing produced homozygous *C9orf72*^*-/-*^ animals. To increase our confidence in the absence of heritable off-target mutations, we sequenced *C9orf72*^*-/-*^ specimens at the three genomic regions most susceptible to off-target cleavage, as predicted by the CRISPRoff tool [28]. These regions included intron 2 of *pou6f1*, intron 3 of *si:dkey-277i15.2*, and exon 11 of *ch25hl3*. Sequencing revealed no evidence of off-target cleavage, as all regions were identical to those in *C9orf72*^+/+^ controls (**S1 Fig****).**

To validate the LOF in our *C9orf72* KO line, we performed reverse transcription-quantitative polymerase chain reaction (RT-qPCR) using zebrafish-specific TaqMan probes targeting *C9orf72* mRNA. We observed a significant reduction in *C9orf72* mRNA levels in 2 days post-fertilization (dpf) larvae: 55.8 ± 3.5% in *C9orf72*^*-/+*^ and 34.7 ± 4.5% in *C9orf72*^*-/-*^ compared to *C9orf72*^*+/+*^ wild-type controls (set at 100%; *p = 0.0041 and* p *< 0.0001,* respectively) (**Fig 1D**). These results suggest that the frameshift mutation likely triggers nonsense-mediated decay [29], confirming that the introduced indels effectively induce *C9orf72* LOF. To further validate the *C9orf72* KO line, we performed Western blot analysis. As the only previously reported antibody for zebrafish C9orf72 protein (Novus Npb2–15656) [18] had been discontinued, we screened eight antibodies known to recognize mammalian C9orf72 [30]. Of these, only Abcam ab221137 and GeneTex GTX634482 detected zebrafish C9orf72, with Abcam ab221137 demonstrating superior specificity (**S2 Fig**). Using ab221137, reported to target the N-terminal segment of the protein, we confirmed the complete absence of C9orf72 in both adult and 2 dpf *C9orf72*^-/-^ specimens (**Fig 1E**). Together, these findings demonstrate that our genetic approach successfully abolishes C9orf72 protein production *in* *vivo*. Thus, this *C9orf72* KO line can be used as a tool to investigate the role of *C9orf72* LOF in ALS and its fundamental biological functions.

### Phenotypic characterization of *C9orf72* knockout zebrafish larvae

**Fig 2:**
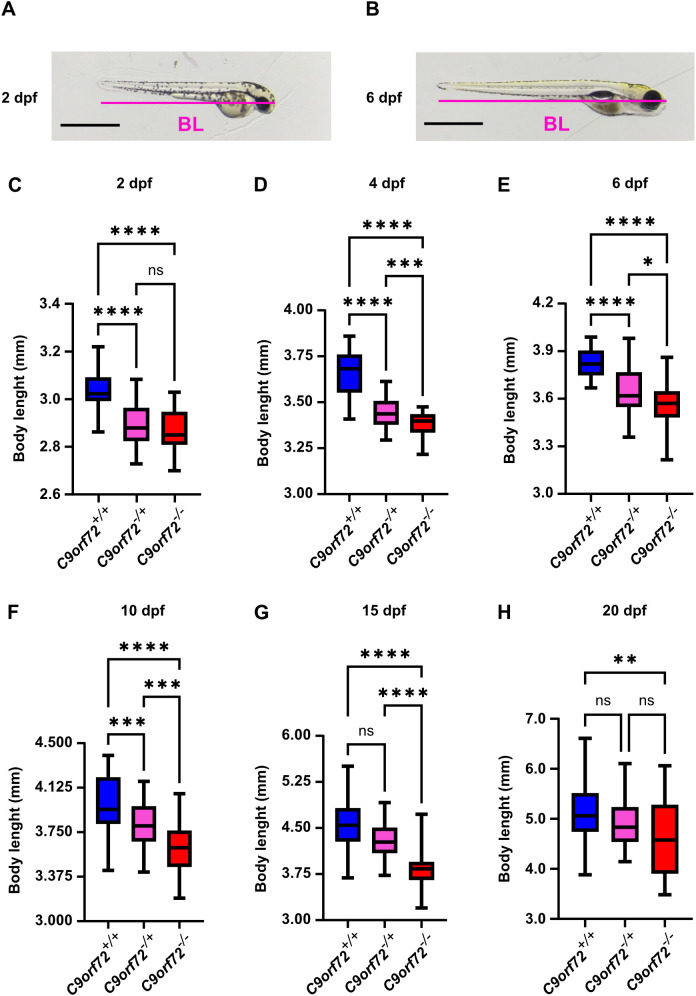
C9orf72 knockout results in persistent size reduction during late embryonic and larval stages. **(A)** Schematic representation of a *C9orf72*^*+/+*^ zebrafish larvae body length (BL) measurement at 2 and 4 days post-fertilization (dpf). **(B)** Schematic representation of a *C9orf72*^*+/+*^ specimen BL measurement from 6 dpf onward. **(C-H)** Body length analysis at 2, 4, 6, 10, 15, and 20 dpf of *C9orf72*^*+/+*^, *C9orf72*^*-/+,*^ and *C9orf72*^*-/-*^ specimens. **(C)** Significant size reduction observed for *C9orf72*^*-/+*^ and *C9orf72*^*-/-*^ specimens compared to *C9orf72*^*+/+*^ controls at 2 dpf. **(D-F)** The *C9orf72*^*-/+*^ and *C9orf72*^*-/-*^ specimens remain significantly smaller than *C9orf72*^*+/+*^ controls at 4, 6, and 10 dpf, with *C9orf72*^*-/-*^ specimens also significantly shorter than *C9orf72*^*-/+*^ at these time points. **(G)** At 15 dpf, *C9orf72*^*-/-*^ specimens are significantly smaller than both *C9orf72*^*+/+*^ and *C9orf72*^*-/+*^. **(H)** At 20 dpf, *C9orf72*^*-/-*^ specimens stay significantly smaller than *C9orf72*^*+/+*^ controls. Statistical tests: ordinary one-way ANOVA with Tukey#39;s multiple comparisons post-hoc test **(C, F)**; Welch and Brown-Forsythe ANOVA with Dunnett#39;s T3 multiple comparisons post-hoc test **(D, E)**; Kruskal-Wallis test with Dunn#39;s multiple comparisons post-hoc test **(G, H)**. **** *p  <  0.0001*, *** *p ≤ 0.001*, ** p *<  0.01*, * *p ≤ 0.05,* and NS *p > 0.05*. Boxplot extremities indicate maximum and minimum values; box limits show the interquartile range (central 50%), and the central line marks the median value. n = 35–45 individual larvae per genotype. Scale bars = 1 mm.

No overt morphological abnormalities were observed in *C9orf72*^*-/+*^ or *C9orf72*^*-/-*^ specimens during embryonic development (0–3 dpf) or up to 20 dpf, aside from a size reduction in *C9orf72*^*-/+*^ *and* C*9orf72*^*-/-*^ specimens (**Fig 1F** and Fig 2A-B). However, C9orf72 depletion resulted in a significant (*p < 0.0001*) decrease in survival from 10 dpf onward, compared to wild-type controls. Survival rates at 20 dpf were 89 ± 1.8%, 76.7 ± 2.4%, and 35 ± 2.8% for *C9orf72*^*+/+*^, C*9orf72*^*-/+,*^ and *C9orf72*^*-/-*^ specimens, respectively (Fig 1G). Additionally, we observed a persistent and statistically significant reduction in body length (BL) from 2 to 12 dpf in both *C9orf72*^*-/+*^ and *C9orf72*^*-/-*^ specimens, compared to *C9orf72*^*+/+*^ larvae (Fig 2A-B). This growth deficit was dose-dependent, persisting until 20 dpf specifically in *C9orf72*^*-/-*^ specimens, which exhibited a more pronounced and consistent reduction across all measured time points (Fig 2C-H, **S1 Table**).

**Fig 3:**
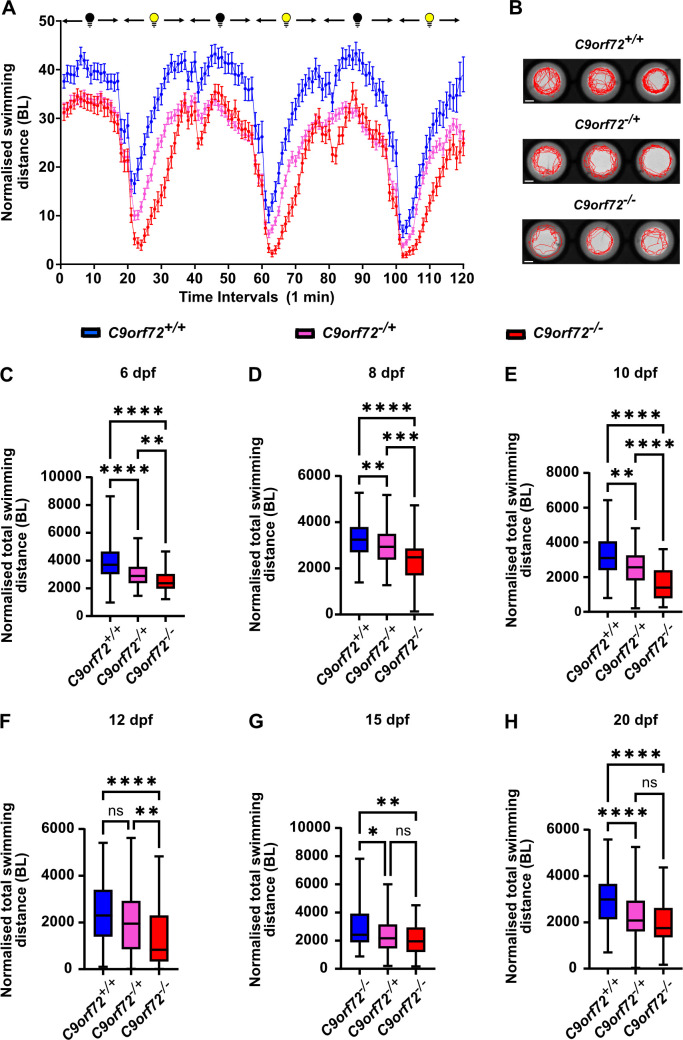
The knockout of *C9orf72* leads to a sustained decrease in swimming activity in larvae. **(A)** Typical swimming distance normalized by mean body length (BL) per minute pattern observed during a 120-minute alternating dark-light paradigm, consisting of 20-minute periods, for *C9orf72*^*+/+*^, *C9orf72*^*-/+,*^ and *C9orf72*^*-/-*^ 6 days post-fertilization (dpf) larvae. Data are presented as mean  ±  SEM. (**B)** Representative 60-second swimming tracks of *C9orf72*^*+/+*^, *C9orf72*^*-/+,*^ and *C9orf72*^*-/-*^ 6 dpf zebrafish larvae subjected to the phasic program. (**C-H)** Quantitative analysis of total swimming distance normalized by corresponding genotype mean BL at 6, 8, 10, 12, 15, and 20 dpf after 120 minutes of exposure to our phasic program. **(C-E)** A significant deficit in swimming activity is observed for C9orf72^-/+^ and *C9orf72*^*-/-*^ compared to *C9orf72*^*+/+*^ controls, and for *C9orf72*^*-/-*^ compared to *C9orf72*^*-/+*^ specimens at 6, 8, and 10 dpf. **(F)** *C9orf72*^-/-^ specimens show a significant deficit in normalized swimming activity compared to *C9orf72*^*+/+*^ and *C9orf72*^*-/+*^ larvae at 12 dpf. **(G-H)** *C9orf72*^*-/-*^ and *C9orf72*^*-/+*^ specimens exhibit a significant reduction in normalized swimming activity compared to *C9orf72*^*+/+*^ controls at 15 and 20 dpf. Statistical tests: Kruskal-Wallis test with Dunn#39;s multiple comparisons post-hoc test (**C, E, F, G, H**), Brown-Forsythe ANOVA with Dunnett#39;s T3 multiple comparisons post-hoc test **(D)**. **** *p  <  0.0001*, ** *p ≤ 0.01*, * *p ≤ 0.05,* and NS *p > 0.05*. Boxplot extremities indicate maximum and minimum values, while box limits represent the range of the central 50% of the data, and the central line marks the median value. N = 3, n = 72 for each genotype except for *C9orf72*^*-/+,*^ where n = 144. N represents the number of experimental repeats from different clutches, and n signifies the total number of larvae per genotype considered for the assay. Scale bars  =  0.2 cm.

To assess the impact of *C9orf72* LOF on motor function, we analyzed zebrafish larval behavior at multiple developmental stages (6, 8, 10, 12, 15, and 20 dpf). As zebrafish larvae exhibit highly stereotyped swimming behaviors even under alternating light/dark conditions [31], we employed a 120-minute alternating dark-light paradigm to assess locomotor activity (Fig 3A**-B**). This paradigm integrates both autonomous light/dark swimming behavior and acute responses to illumination changes. As larval size influences swimming performance, total locomotor activity was normalized to mean (BL) for each genotype at the corresponding age. Our analysis revealed a significant and persistent locomotor deficit in *C9orf72*^*-/+*^ and *C9orf72*^*-/-*^ larvae compared to *C9orf72*^*+/+*^ controls at 6, 8, 10, 15, and 20 dpf (Fig 3C**-H**). The locomotor deficit was dose-dependent, with *C9orf72*^*-/-*^ larvae exhibiting a more severe impairment compared to *C9orf72*^*-/+*^ larvae until 15 dpf. Notably, the most pronounced motor differences among genotypes, especially between *C9orf72*^*-/+*^ and *C9orf72*^*-/-*^ larvae, typically occurred during the early recovery phase following the dark-to-light transition (**Fig 3A**, **S3 Fig**). The *C9orf72* LOF-dependent locomotor deficit was also evident under a 2-phase paradigm (20-minute dark / 60-minute light; (**S4 Fig**)) and persisted even when only considering the stable spontaneous swimming period (SSAP) (**S5 Fig**). The SSAP was defined as the 40–80 min window, following the 20–40 min adaptation phase after the light transition.

**Fig 4:**
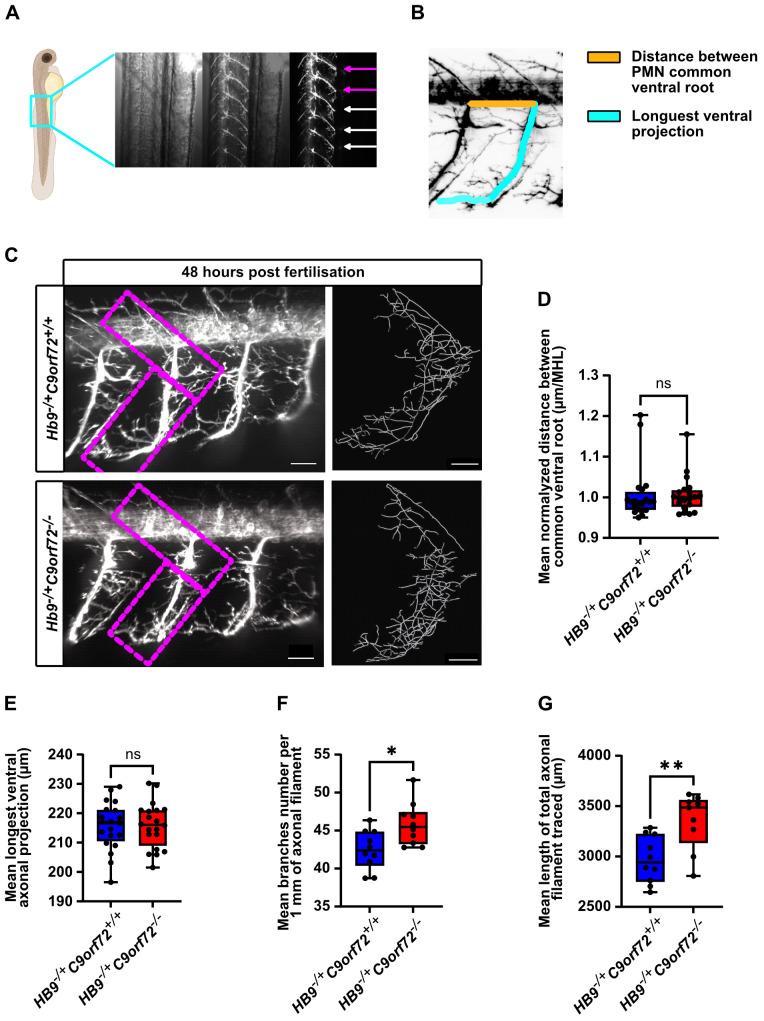
Complete loss of C9orf72 leads to mild axonal over-branching of spinal primary motor neurons. **(A)** Schematic representation of the dorsal primary motor neuron (PMN) units analyzed for measurements of the common ventral root distance (VRD), longest ventral axonal filament (LVF), and 3D axonal filament tracing in *Hb9*^*-/+*^*C9orf72*^*+/+*^ and *Hb9*^*-/+*^*C9orf72*^*-/-*^ larvae at 48 hours post-fertilization (hpf). **(B)** Schematic depiction of VRD and LVF measurements, illustrating how the distance between the common PMN’s ventral root and the longest projecting axonal filament were quantified. **(C)** Representative confocal image of a spinal hemisegment PMN unit and corresponding 3D axonal filament tracing used to assess axonal branching. Dashed lines indicate the area considered for filament tracing. **(D, E)** Quantitative analysis of mean VRD **(D)** and mean LVF **(E)** revealed no significant differences between *Hb9*^*-/+*^*C9orf72*^*+/+*^ and *Hb9*^*-/+*^*C9orf72*^*-/-*^ larvae. To account for size differences, mean VRD was normalized to the mean hemisegment length (MHL) of the five PMN considered for each specimen (N = 20, n = 100). **(F, G)** Quantitative analysis of axonal branching showed a mild but significant increase in the mean number of branches per 1 mm of axonal filament **(F)** and a significant increase in mean total traced axonal filament length **(G)** in *Hb9*^*-/+*^*C9orf72*^*-/-*^ larvae compared to controls. Two PMNs were traced per specimen per genotype (N = 9-10, n = 18-20). *Statistical tests:* Mann-Whitney test **(D);** unpaired two-tailed parametric t-test **(E–G**). ** p ≤ 0.01, * p ≤ 0.05, NS p > 0.05. Boxplot representation: Extremities indicate minimum and maximum values; box limits represent the interquartile range (central 50%), and the central line marks the median. N represents the number of distinct specimens; n represents the total number of PMN units per genotype analyzed. Scale bars = 30 µm.

To identify potential neuroanatomical causes underlying the motor deficits observed between 6 and 20 dpf in *C9orf72* KO larvae, we examined the morphological features of spinal primary motoneurons (PMNs). In zebrafish, PMNs are large, segmentally organized MNs located in the spinal cord, projecting ventrally and laterally to innervate the ventral trunk musculature within each spinal hemisegment [32]. To visualize PMNs *in vivo*, we crossed *C9orf72*^*-/-*^ specimens with transgenic *Hb9:GFP* zebrafish (*Tg(mnx1:GFP)*), generating a stable *Hb9C9orf72*^*-/-*^ line. The *Mnx1* promoter drives green fluorescent protein (GFP) expression primarily in motoneurons, allowing us to assess the morphology of spinal PMNs at 48 hours post-fertilization (hpf) in embryos embedded in low-melt agarose (Fig 4A). To determine whether C9orf72 KO affected PMNs organization, we first measured the distance between the common PMNs ventral root and the longest ventral axon projection of the first five hemisegments caudal to the yolk (Fig 4A-B). The distance between PMNs ventral roots was unaffected by *C9orf72* KO at 48 hpf, as was the longest ventral projection length (Fig 4D-E). We next investigated whether *C9orf72* KO induced more subtle morphological defects in PMNs of larvae. To do so, we manually traced the axonal filaments of PMNs in 3D within the first two hemisegments caudal to the yolk (Fig 4A, C), starting at the common axonal ventral root of PMNs. Our analysis revealed that *Hb9*^*-/+*^*C9orf72*^*-/-*^ larvae exhibited mild axonal disruption in PMNs, characterized by a slight but statistically significant increase in axonal branching at 48 hpf compared to *Hb9*^*-/+*^*C9orf72*^*+/+*^ controls (*p = 0.0118*; 45.83 ± 0.81 vs. 42.46 ± 0.89 branches per millimeter of filament) (Fig 4F). Additionally, *C9orf72* KO specimens displayed a significantly greater total axonal filament length in the region of interest (*p  =  0.0047*; 3354 ± 93.84 µm vs. 2970 ± 73.38 µm).

To further investigate potential neurological defects underlying the motor deficits observed in *C9orf72* KO larvae, we examined NMJ integrity in spinal hemisegments. We performed double immunohistochemistry on 6 dpf larvae, using an SV2 antibody to mark presynaptic terminals and α-bungarotoxin (α-BTX) to label postsynaptic acetylcholine receptors (AChR). Quantitative analysis revealed a significant increase (Ordinary one-way ANOVA, *p < 0.001*) in the total number of postsynaptic α-BTX puncta in *C9orf72*^*-/-*^ larvae (4752 ± 133.4 puncta) compared to *C9orf72*^*+/+*^ (3958 ± 164.5 puncta) and *C9orf72*^*-/+*^ specimens (3842 ± 149.3 puncta) (S6 Fig **B**). However, there was no significant difference in the total number of presynaptic SV2 puncta (S6 Fig C). Additionally, analysis of presynaptic and postsynaptic signal overlap at the NMJ using two alignment thresholds (100% and 50%) revealed no significant differences between genotypes, indicating preserved synaptic colocalization. (**S6 Fig** D-G). These findings suggest that complete loss of C9orf72 selectively alters the postsynaptic compartment at the NMJ, while overall synaptic architecture and alignment remain unaffected.

### Therapeutic compound screening and validation

**Fig 5:**
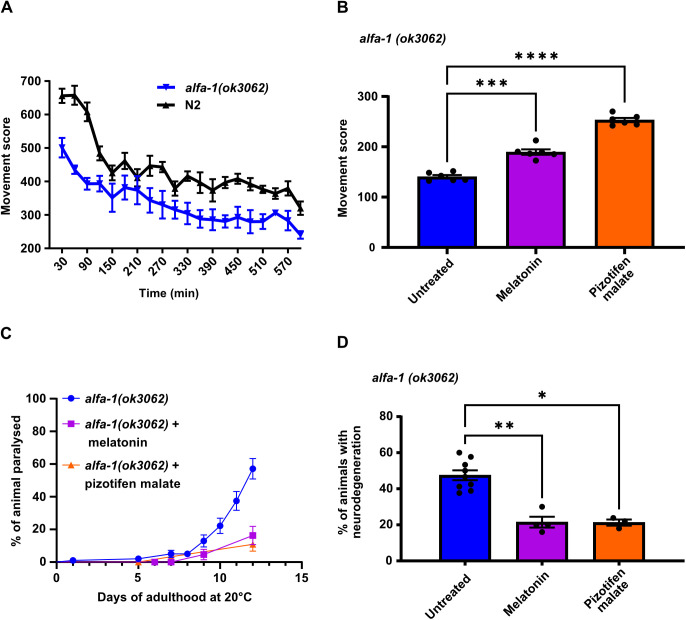
Pizotifen malate and melatonin ameliorate deleterious phenotypes in *C. elegans* knockout *for C9orf72* orthologue (*alfa-1*). **(A-B)** Motor activity of *alfa-1(ok3062)* and N2 (wild-type) worms measured in liquid medium using automated infrared beam scattering. **(A)** *alfa-1(ok3062)* worms exhibit motility impairment after 30 minutes compared to N2 controls, which persists throughout the 600 minutes (10-hour) assay. Data are presented as mean ± SEM; n = 3 technical replicates. **(B)** Improvement in motor activity of *alfa-1(ok3062)* worms treated with melatonin or pizotifen malate (PM) compared to untreated controls. Motor activity was measured in 30-minute bins over 180 minutes and expressed as a movement score. Data are presented as mean ± SEM; N = 3 technical replicates, n = 6 repeated measurements per replicate. Two-way ANOVA with Dunnett#39;s post-hoc test. *** *p < 0.001*, **** *p < 0.0001*. **(C)** Kaplan-Meier survival analysis of age-dependent paralysis in *alfa-1(ok3062)* worms on solid media containing 1% DMSO (untreated), melatonin, or PM. Both compounds significantly delay paralysis compared to untreated controls. *p < 0.001* (log-rank Mantel-Cox test); N = 3 plates, n = 60-99 worms per condition. **(D)** Percentage of *alfa-1(ok3062)* worms displaying neurodegeneration at 9 days post-adulthood on solid media containing 1% DMSO (untreated), melatonin, or PM. Both compounds significantly reduce neurodegeneration. Kruskal-Wallis test with Dunn’s multiple comparison post-hoc test. * *p < 0.05*, ** *p < 0.01*; N = 3-9 worm synchronisation, n = 90-270 worms per condition. Data are presented as mean ± SEM.

The motor deficits observed in our *C9orf72* KO zebrafish model represent an ALS-related phenotype suitable for therapeutic compound screening. However, zebrafish are less amenable to high-throughput screening than *C. elegans*. Therefore, large compound libraries can first be screened in *C. elegans* utilizing ALS-related phenotypes, with candidates refined through validation of additional phenotypes and the most promising hits tested in our zebrafish model. Notably, *C. elegans* display a stereotyped swimming motion in liquid medium that actively engages the NMJ, making it an effective model for assessing MN health [33]. Additionally, high-throughput liquid culture assays using automated infrared beam scattering have been developed to measure locomotor activity and evaluate the neuroprotective effects of compounds in *C. elegans* [34]. Given that *alpha-1(ok3062)* mutants exhibit MN degeneration and NMJ dysfunction, we investigated their locomotor phenotype in liquid culture to confirm their suitability for high-throughput screening. After 30 minutes, young adult *alpha-1(ok3062)* mutants showed significantly reduced swimming activity compared to wild-type (N2) worms (*p = 0.0015*, Fig 5A). This deficit persisted for up to 600 minutes (10-hour) (Fig 5A), making it suitable for high-throughput compound screening, and was statistically significant for all but one of the 30-minute intervals. We screened over 4,000 bioactive compounds from commercially available drug libraries using a high-throughput liquid assay, identifying 80 compounds that significantly enhanced locomotion in *alpha-1(ok3062)* mutants (*p <* *0.05*, S4 Table). From the 80 primary hits, the 12 compounds showing the most significant and consistent improvement in locomotion in *alpha-1(ok3062)* mutants were selected for further validation on solid media. All 12 compounds significantly improved age-dependent paralysis and neurodegeneration phenotypes in *alpha-1(ok3062)* mutants (*p < 0.05*, S5 Table).

Among the 12 compounds, we selected melatonin and pizotifen malate (PM) for validation in zebrafish based on robust and consistent efficacy across the *C. elegans* assays, feasibility for waterborne exposure in zebrafish larvae (including solubility and stability), and prior precedent for use in zebrafish larvae at comparable concentration ranges [35,36]. Both compounds improved locomotor deficits, with treated mutant worms showing significantly greater locomotor activity (melatonin: 189.4 ± 5.3 movement score units; PM: 253.1 ± 4.3 movement score units) compared to untreated controls (140.4 ± 3.4 units; *p <* *0.001* for both, **Fig 5B**). They also increased the proportion of *alfa-1(ok3062)* mutants unaffected by age-related paralysis at day 12 (melatonin: 83.7 ± 5.6%; PM: 89.1 ± 4.3%) compared to untreated controls (42.9 ± 6.3%; *p < 0.001* for both, **Fig 5C**). Neurodegeneration at day 9 was also significantly reduced in treated *alfa-1(ok3062)* worms (melatonin: 21.3 ± 3.0%; PM: 21.27 ± 1.3%) compared to untreated controls (47.5 ± 2.7%; *p = 0.0078* and *p = 0.0313*, respectively, **Fig 5D**). Therefore, we aimed to validate the therapeutic potential of melatonin and PM for *C9orf72* LOF-related phenotypes in zebrafish. To do this, we assessed their ability to alleviate swimming deficits seen in *C9orf72* LOF larvae.

**Fig 6:**
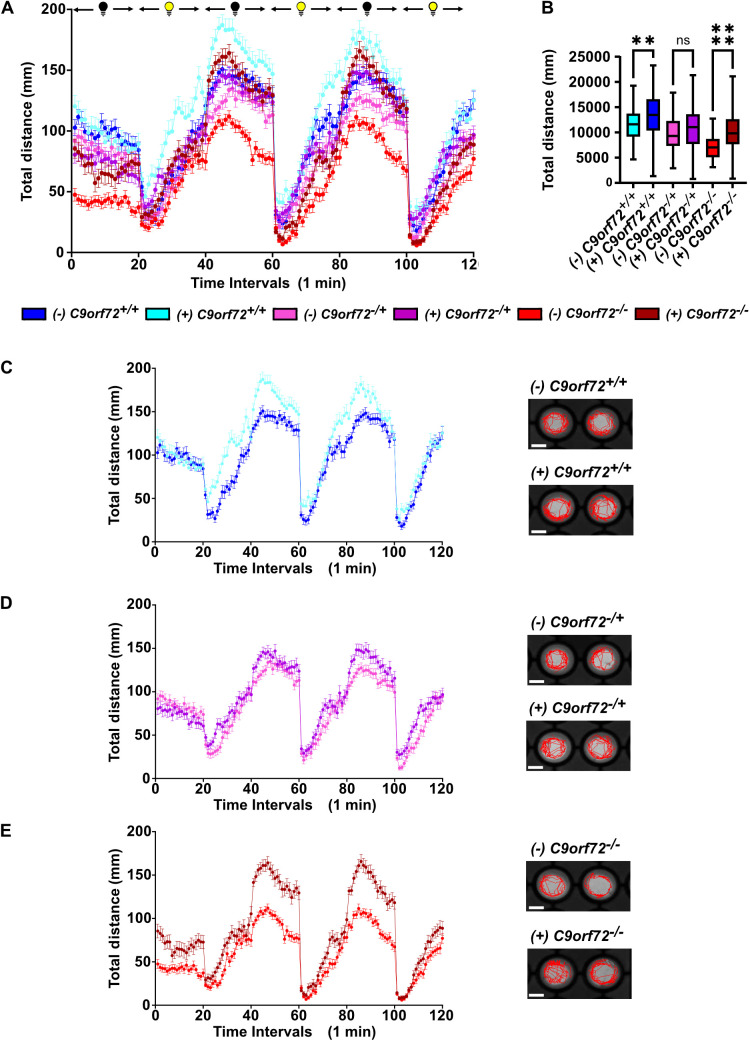
Pizotifen malate increases total swimming activity and partially rescues locomotor deficits in *C9orf72* knockout larvae. **(A)** Mean swimming distance per minute for pizotifen malate 0.5 µM (PM) treated (+) and untreated (-) *C9orf72*^*+/+*^, *C9orf72*^*-/+*^, and *C9orf72*^*-/-*^ six day post-fertilization (dpf) zebrafish larvae under a 120-minute phasic dark-light program. Data are presented as mean ± SEM. **(B)** Quantitative analysis of total swimming distance shows significant increases in PM-treated (+) *C9orf72*^*+/+*^ and *C9orf72*^*-/-*^ larvae compared to untreated (-) controls. Data are presented as mean ± SEM. Welch and Brown-Forsythe ANOVA with Dunnett#39;s T3 multiple comparisons post-hoc test. **** *p < 0.0001*, ** *p ≤ 0.01*, NS *p > 0.05*. Boxplot extremities indicate minimum and maximum values; box limits represent the central 50% of the data, and the central line marks the median. **(C-E)** Swimming distance per minute and representative 60-second swimming tracks for treated and untreated *C9orf72*^*+/+*^ **(C)**, *C9orf72*^*-/+*^ **(D**), and *C9orf72*^*-/-*^ **(E)** larvae. Data are presented as mean ± SEM. N = 5, experimental repeats; n = 80, larvae per genotype. Scale bars = 0.2 cm.

We exposed 6 dpf *C9orf72* KO zebrafish larvae to melatonin or PM through in-water dosing that started 2 hours post-fertilization, and we measured their swimming activity under a 120-minute light-dark paradigm (Fig 6A). While various concentrations of melatonin (1, 0.5 and 0.25 µM) did not significantly improve motor activity in any of the *C9orf72* LOF genotypes (*p > 0.05*, S7A-C Fig), PM treatment at a relatively low concentration (0.5 µM) significantly enhanced total swimming activity in *C9orf72*^*-/-*^ larvae (7003 ± 255 mm vs. 10,380 ± 438 mm; p < 0.0001, Fig 6B, E). PM also increased total swimming activity in wild-type (*C9orf72*^*+/+*^) larvae (11,592 ± 379 mm vs. 13,628 ± 541 mm; p = 0.0075, Fig 6B, C). In contrast, the increase in total swimming activity for *C9orf72*^*-/+*^ larvae due to PM exposure was not statistically significant (Fig 6B, D). The extent of PM-induced increase in locomotion was greater *in C9orf72*^*-/-*^ larvae (+38.9 ± 0.06%) than in wild-type larvae (+16.2 ± 0.05%). The effects of PM were especially pronounced during the dark phases, with generally weaker effects during the light phases (Fig 6C-E). Conversely, exposure to higher concentrations of PM (1, 2.5, and 5 µM) had no significant effect on the activity of *C9orf72*^*-/-*^ larvae at lower doses (S7D–F Fig), and exposure at 5 µM significantly reduced total swimming activity in both *C9orf72*^*-/-*^ and *C9orf72*^*-/+*^ larvae (p *≤* 0.05, S7D–E Fig).

## Discussion

### Insights from a new *C9orf72* loss-of-function zebrafish model

*C9orf72* hexanucleotide repeat expansions are the most common genetic cause of ALS/FTD. It remains unclear whether *C9orf72* LOF, DPR production, and RNA foci gain-of-function, or a combination of those mechanisms, is causative for the disease. Here, we generated and characterized a new stable *C9orf72* KO zebrafish line, providing a promising vertebrate model to investigate *C9orf72* LOF and validate potential therapeutic compounds through its *C9orf72*-related ALS phenotypes. The *C9orf72*^*-/+*^ and *C9orf72*^*-/-*^ mutants exhibited motor deficits, growth delays, and increased mortality from early to late larval stages. *C9orf72*^*-/-*^ larvae also showed mild overbranching and increased axonal filament length in spinal PMNs, along with subtle NMJ disruptions characterized by increased postsynaptic acetylcholine receptor (AChR) density. Although our results support a functional contribution of *C9orf72* LOF to early neuromuscular phenotypes, the broader physiological role(s) of C9orf72 and the precise molecular and cellular mechanisms underlying these effects remain to be fully resolved.

The increased axonal branching and filament length in *Hb9*^*-/+*^*C9orf72*^*-/-*^ larvae may reflect early neurodevelopmental dysregulation rather than degeneration, consistent with the absence of motor neuron loss at this stage. Similar phenotypes in ALS-linked models (e.g., SOD1, SMA) are thought to arise from aberrant axon guidance or compensatory sprouting [37–39]. In C9orf72-deficient systems, impaired vesicle release, autophagy, or trafficking may underlie these changes [18,40–42]. Though axonal overbranching is not reported in C9-ALS patients, early synaptic and transport deficits are well documented [43], suggesting that C9orf72 loss could destabilize motor circuits and increase susceptibility to later insults [44]. These findings underscore C9orf72’s role in maintaining circuit precision and highlight the need for longitudinal or combinatorial studies.

The selective increase in postsynaptic AChR density, without corresponding changes in presynaptic SV2 puncta or synaptic alignment, likely reflects an early compensatory or maladaptive response to impaired neurotransmission that emerges under near-complete C9orf72 LOF. C9orf72 has been implicated in synaptic vesicle trafficking, autophagy, and neurotransmitter release [18,40,41,43,45] and its loss may indirectly influence AChR abundance or stability at the NMJ through homeostatic plasticity mechanisms [46]. Comparable NMJ structural remodelling occurs in ALS models such as the SOD1-G93A mouse model, where postsynaptic AChR reorganization precedes overt denervation [47,48], and related synaptic alterations in both NMJ and CNS contexts have also been reported in other C9orf72-deficient systems [18,45]. These findings suggest early NMJ vulnerability due to *C9orf72* LOF and underscore the need for future electrophysiological analyses to assess functional synaptic consequences.

As our study focuses on larval stages, we did not perform any formal analyses in adult fish aside from assessing C9orf72 expression. Nonetheless, *C9orf72*^*-/-*^ specimens developed normally into adulthood, surviving and reproducing for up to 12 months without significant health deterioration. These findings support a role for C9orf72 deficiency in the manifestation of *C9-*ALS related phenotypes, while also indicating that C9orf72 LOF alone may be insufficient to induce overt neurodegenerative phenotypes during early larval stages in zebrafish. Future studies should extend behavioral and neurodegenerative assessments into adulthood and aging to determine whether the early larval phenotypes observed here progress to later ALS-relevant pathology.

### Comparison to previous *C9orf72* loss-of-function zebrafish models

RNAi-based KD of *C9orf72* in zebrafish larvae has been shown to cause severe overbranching and truncation of caudal primary MNs [17], as well as motor deficits, morphological abnormalities, reduced survival, and disrupted NMJ integrity [18]. In contrast, our *C9orf72*^*-/+*^ larvae displayed no overt morphological defects, only a mild reduction in survival, and a persistent but less pronounced decrease in motor activity. The *C9orf72*^*-/-*^ specimens exhibited early mild overbranching of spinal PMN axons, with longer axonal filaments primarily due to increased branching and a greater number of postsynaptic structures. While *C9orf72*^*-/-*^ specimens exhibited more pronounced survival and motor deficits than *C9orf72*^*-/+*^ specimens, these phenotypes were still milder than those reported in the microRNA-based *C9orf72 KD* model. Unlike RNAi-based approaches, our zebrafish *C9orf72* KO model incorporates a stable genomic mutation, reducing susceptibility to off-target effects but potentially triggering compensatory genetic responses via nonsense-mediated decay [49]. Additionally, it enables the investigation of complete C9orf72 loss effects. The phenotypic discrepancies between our *C9orf72*^*-/+*^ specimens and the RNAi KD models highlight the challenges in interpreting KD-based findings without validation in a stable LOF mutant.

The comparatively mild phenotypes observed in our stable zebrafish C9orf72 LOF model may, in part, reflect compensatory genetic responses that arise in germline mutants during development [50,51]. In zebrafish and other systems, stable LOF alleles can induce transcriptional adaptation and related compensatory mechanisms, reducing phenotypic penetrance relative to RNA-level KD approaches [50–52]. Consistent with this, systematic analyses in zebrafish have shown that antisense morpholino KD phenotypes often do not recapitulate corresponding mutant phenotypes, underscoring method-dependent differences between KD and stable LOF models [52]. Such compensation may contribute to the milder structural and behavioral abnormalities observed in our C9orf72 LOF larvae despite substantial C9orf72 deficiency and could also influence genotype-dependent pharmacological responses, although alternative explanations cannot be excluded. Together, these considerations highlight the importance of developmental context, allele type, and the mode and degree of gene perturbation when interpreting LOF phenotypes.

The recently reported CRISPR/Cas9-generated *C9orf72* KO zebrafish model supports several of our findings. Notably, they observed an absence of both widespread NMJ alterations and MN degeneration in the spinal cord of their adult specimens [19]. Our *C9orf72* KO model does not phenotypically conflict with the previously reported KO model, although there is limited overlap in the scope and nature of the characterization approaches. A key distinction between the two KO models is the predicted truncated C9orf72 protein, which is 29% of the full-length sequence in their model compared to 15% in ours, resulting from a premature stop codon downstream of our mutation sites. Furthermore, we confirmed complete C9orf72 protein ablation in *C9orf72*^*-/-*^ specimens using a known cross-reactive antibody for human and mouse, which we validated for zebrafish C9orf72, whereas their model demonstrated reduced *C9orf72* mRNA levels [19]. As our study relies on a single CRISPR-derived allele without complementation or rescue, allele-specific or off-target effects cannot be fully excluded, despite multi-generational backcrossing and off-target site analysis. While our molecular data support its classification as a likely null, the possibility that it is hypomorphic or encodes a dominant-negative truncated product remains. Further studies, such as transgenic rescue or epitope mapping, will be needed to clarify the mutation’s precise nature and mechanism.

### Visual and circadian rhythm impairment as possible confounding factors

Jaroszynska et al. (2024) [19] reported retinal degeneration and gliosis in 24-month-old *C9orf72*^*-/-*^ zebrafish, indicating potential visual impairment. However, we argue that this effect is minimal during larval stages, as they found no retinal abnormalities at 5 days post-fertilization (dpf), and only limited inner nuclear layer neuron loss was identified by 8 months, with the photoreceptors remaining unaffected. Additionally, locomotor deficits in *C9orf72*^*-/-*^ and *C9orf72*^*-/+*^ larvae continued even under conditions that reduce the impact of acute light stimuli, such as our biphasic dark-light paradigm and locomotor assessment conducted only after light adaptation (SSAP). Furthermore, a zebrafish model of moderate perceptual alteration (hyperopia) showed no changes in spontaneous swimming distance at 7 dpf under consistent light or dark conditions [53]. Although our study was not designed to assess circadian rhythms, the persistent locomotor deficits observed across both light and dark phases suggest that these impairments are unlikely to reflect circadian misalignment. However, previous studies have reported altered circadian activity patterns in *C9orf72* KO mice, particularly during the dark (active) phase [9]. Future studies could investigate circadian gene expression, free-running activity, and rest–wake cycles in C9orf72-deficient zebrafish to determine whether circadian mechanisms contribute to behavioral phenotypes. Altogether, these findings suggest that neuromuscular disruptions or other *C9orf72* LOF effects, rather than visual impairment or circadian disruption, primarily drive the locomotor phenotype in our zebrafish model.

### Pizotifen malate as a potential therapeutic compound for ALS

We demonstrated that *alfa-1(ok3062) C. elegans* exhibit early motor deficits in liquid, which are suitable for automated high-throughput compound screening. This enabled us to identify 80 compounds that improved the locomotor deficits, with 12 also demonstrating the ability to alleviate age-dependent paralysis and neurodegeneration. Among the 12, melatonin and PM were selected for further phenotypic compound validation in *C9orf72* LOF zebrafish larvae by evaluating their persistent locomotor deficiencies. Locomotion assays revealed that low-dose PM (0.5 µM) significantly improved swimming deficits in *C9orf72*^*-/-*^ larvae at 6 days post-fertilization, while melatonin did not show significant effects across genotypes or tested concentrations.

Although melatonin improved ALS-related phenotypes in *alfa-1(ok3062) C. elegans* specimens, it did not measurably rescue locomotor deficits in our *C9orf72* LOF zebrafish larvae under the conditions tested. This discrepancy could reflect species- and developmental context–dependent differences in melatonin responsiveness rather than an absence of biological activity. In vertebrate systems, melatonin’s effects are frequently linked to circadian regulation, oxidative stress modulation, or neuroprotection in mature or stressed neural circuits [54], which may be less engaged during early larval zebrafish locomotor behavior. In addition, the dose range compatible with behavioral assays in zebrafish larvae is constrained by the sedative or activity-suppressing effects of melatonin at higher concentrations, potentially limiting the detection of beneficial effects under our experimental conditions [55].

PM is approved for human use in Canada [56], France [57], and the UK [58]. It primarily acts as a 5-HT₂ serotonin receptor antagonist with weak anticholinergic, H1 antihistamine, and antikinin activity. Historically, PM has been utilized for preventing vascular headaches [59] and treating depressive disorders [60]. Given the existence of zebrafish orthologues for 5-HT₂ [61] and H1 receptors [62], higher PM doses were anticipated to induce mild sedation, consistent with findings in rodents and primates [59]. At 5 µM, PM exposure significantly decreased locomotion in *C9orf72*^*+/+*^ and *C9orf72*^*-/+*^ larvae but did not affect locomotion in *C9orf72*^*-/-*^ larvae, whereas 0.5 µM surprisingly increased locomotion in *C9orf72*^*+/+*^ larvae. Interestingly, *C9orf72*^-/+^ larvae did not show significant improvement at any PM dose tested, suggesting that partial C9orf72 loss may trigger compensatory adaptations that blunt pharmacological responsiveness.

These genotype-specific differences highlight the importance of neuromodulatory context.PM’s rescue of paralysis in *C. elegans* and motor deficits in zebrafish *C9orf72*^*-/-*^ larvae suggests neuroprotective potential, though the precise mechanism remains uncharacterized. This may reflect altered neurotransmitter release, receptor sensitivity, or impaired homeostatic buffering in *C9orf72*^*-/-*^ larvae, rendering them more responsive to serotonergic modulation. This genotype-dependent effect is reflected in both the Cohen’s d (1.36 vs. 0.68) and fold-change in locomotion (1.482 vs. 1.176), supporting a stronger therapeutic response in mutants. While PM likely acts via broad neuromodulatory pathways, its selective benefit in *C9orf72*^*-/-*^ zebrafish and neuroprotective action in *C. elegans* suggest disease relevance. Further validation in unrelated ALS models will be critical to assess specificity. Future studies should investigate dose–response dynamics, long-term safety, and underlying circuit-level effects through electrophysiology. These findings are consistent with previous reports of PM reducing neurodegeneration and enhancing motor performance in the R6/2 Huntington’s disease mouse model [63].

## Conclusion

We generated and characterized stable *C9orf72*^*-/+*^ and *C9orf72*^*-/-*^ zebrafish lines, whose phenotypes suggest a link between *C9orf72 loss-of*-function and the manifestation of ALS-related phenotypes. However, they demonstrated significant discrepancies with the known zebrafish RNAi-based *C9orf72* knockdown (KD) models. These lines serve as promising tools for studying C9orf72 function, the synergistic effects of ALS risk factors, mutations, and pathways potentially causative for the pathology, as well as for validating prospective ALS therapeutic compounds in a rapid and cost-effective *in vivo* vertebrate model system. Future longitudinal studies will be needed to determine whether C9orf72 LOF accelerates age-related neurodegeneration in adult zebrafish. We showed that the *C. elegans alfa*-*1(ok3062)* mutants exhibit a locomotor deficit phenotype suitable for high-throughput compound screening, resulting in the identification of 12 compounds that demonstrated neuroprotective effects and improved locomotion. Finally, PM’s ability to alleviate *C9orf72* deficiency-related ALS locomotion phenotypes was demonstrated in the *C9orf72*^*-/-*^ specimens, suggesting its potential for further validation in other ALS models as a prospective therapeutic for the disease. Further validation of PM’s potential to alleviate ALS-related phenotypes, particularly those involving neurodegeneration, in other vertebrate ALS models will be essential for clinical translation.

## Materials and methods

### Ethics statement

All experiments were conducted at the Centre de Recherche du Centre Hospitalier de l#39;Université de Montréal (CRCHUM) in compliance with the Canadian Council for Animal Care (CCAC) guidelines and approved by the Comité Institutionnel de Protection des Animaux (Institutional Animal Care Committee) under protocol number N21017APz. Most experiments were performed on sexually undifferentiated larvae between 2 and 20 dpf, except for tissue extraction on 6-month-old female specimens. The zebrafish experiments and fish lines maintenance were conducted over a period of 6 years from March 15, 2019, to April 1, 2025. Efforts were made to minimize animal suffering and to reduce the number of animals used. This study involved no human derived data.

## Zebrafish husbandry

### System and environmental conditions

Specimens < 5 days post-fertilization (dpf) and all experimental fish except adults used for protein extraction were maintained in E3 medium (5 mM NaCl, 0.17 mM KCl, 0.33 mM CaCl₂, 0.33 mM MgSO₄, 0.00005% w/v methylene blue, pH 7.2) in a dry incubator at 28°C under a 12 h light/dark cycle (lights on 09:00–21:00). Temperature was monitored via the incubator display and a separate thermometer placed in a control solution. Half of the E3 medium was manually replaced daily.

Specimens > 5 dpf used for line maintenance and egg production were housed in a dedicated facility with an Aquaneering aquarium system comprising two linked subsystems (A and B) on a 12 h light/dark cycle (09:00–21:00). Approximately 10% of the water volume was replaced daily via automated backwash using reverse-osmosis-purified water supplemented with marine salts. Water quality (salinity, pH, temperature, conductivity, and system pressure) was continuously monitored and adjusted by a computerized system; deviations triggered an Aquatouch console alarm (Table 1). Water was denitrified by bacterial filtration, mechanically filtered to 50 µm, and sterilized with UV light before redistribution. Water temperature was maintained at 27 ± 0.5°C.

**Table 1:** Key water quality parameters maintained in the system.

Condition	Maintained value
Water temperature	26.5-27.5°C
pH	pH 7.0–7.4
Conductivity	650-750 μS
Fluidized bed biofilter	6-9 psi
Room temperature	24-26°C
*FF1* (Automatic backwash filter pressure-ABF)	15-20 psi
Ammonia	0 ppm
Nitrite	100 μg/L
Nitrate	0-5 ppm or mg/L
Alkalinity	50-150 ppm or mg/L
Water hardness (CaCO_3_)	50-150 ppm
Dissolved oxygen level	>6.0 ppm

### Housing parameters

<5 days post-fertilization specimens were kept in 1-liter containers at a maximum density of ≤50 per 100 mL in 1/3 of the container volume of E3 medium. 5–20 dpf larvae dedicated to experiments were transferred to fresh 1-liter containers every two days and kept at a maximum density of 15–30 specimens per 100 mL. From 10 dpf onward, the E3 medium volume was increased to half the container’s capacity, with daily 50% water changes. For general rearing 5–14 dpf larvae, specimens were transferred to a clean 1-liter plastic container, and the E3 medium was replaced with 1/3 liter of system water. The container was placed in a 2.8-liter tank with system water to maintain the appropriate temperature. Water flow was restricted to the aquarium only. Each day, 25–50% of the system water was replaced with fresh water. From 10 dpf onward, the system water volume in the container was increased to 2/3 of its capacity. Juveniles (≥15 dpf) were transferred to 3-, 6-, or 9.5-liter tanks with mesh filters. Water flow was initiated at two drops per second and doubled daily until a continuous stream was achieved; 25–50% of the water was replaced daily during this transition. Adults (≥90 dpf) were maintained in genotype-specific tanks of 3, 6, 9.5 or 18 liters at a standard density of 4–5 fish per liter for 12–15, 24–30, 38–47 or 72–90 specimens depending on the tank. For breeding, fish were segregated by sex and genotype: short-term matings (≤1 month) used ~12 fish per 2.8-liter tank, while long-term breeding groups were housed at 9 fish per 2.8-liter tank to ensure consistent egg production.

### Feeding protocols

Zebrafish developmental stages relevant to feeding are detailed in **Table 2**, and feeding protocols are summarized in **Table 3**.

**Table 2:** Zebrafish developmental stages for the feeding protocol.

Developmental stage	Age (post-fertilization)
Larvae	5-14 days
Juveniles	14 days to a month
Young adults	1 to 3 months
Adults	≥ 3 months

**Table 3:** Animal facility zebrafish feeding protocols.

Stage	Age (post-fertilization)	Particle size of feed pellets	Distribution
Larvae	5-14 days	GEMMA Micro 75 (50–100 microns)	2-3 times per day (morning, noon, afternoon)
Juvenile	14 days to a month	Shrimps	Morning
		GEMMA Micro 150 (100–200 microns)	3 times (morning, noon, afternoon) per day
Young adults	1 to 3 months	Shrimps	Morning
		GEMMA Micro 300 (200–500 microns)	Once, afternoon
Adults	> 3 months	Shrimps	Morning
		GEMMA Micro 500 (400–700 microns)	Once, afternoon

Opened food and egg containers were stored at 4°C for up to 1 month. Expiration date for dry food diets was 24 months, and 9 months for shrimp diet.

Dry food diets: Dry powder and pellets from the GEMMA Micro product line (Skretting, Norway). Approximately 1/16 teaspoon (~0.31 mL) of dry food pellets was provided per serving for 2.8-liter tanks, 1/8 teaspoon for 6-liter tanks, and ½ teaspoon for the largest tanks (9.5 or 18 liters).

Shrimp diet: *Artemia salina* (Brine shrimp Direct, Utah) were cultured daily in 1-liter of aerated saltwater (20–25 mL sea salt/L) with 20 mL of shrimp eggs, harvested after 42 hours, and dispensed via a 250 mL LDPE wash bottle (3 sec for 2.8-liter tanks, 5–6 sec for 18-liter tanks). All feedings were monitored to ensure consumption within 5 minutes.

Specimens dedicated to experiments: Larvae were fed according to their developmental stage, as described above, with the following adjustments. Larvae and juveniles were fed dry food twice daily: once in the morning before the water change and once in the afternoon. Food amount was adjusted based on specimen number, with ~ 1/16 teaspoon for 100 specimens at 5–10 dpf, and ~ 1/8 teaspoon for 100 specimens at 11–20 dpf.

### Welfare considerations

Housing: Zebrafish were maintained in social groups of ≥3 per 2.8-liter tank to promote natural shoaling behavior, with isolation limited to necessary procedures (genotyping, recovery, or founder identification). Density was adjusted if aggressive behaviors were observed. Environmental enrichment included artificial plants and gravel images in adult tanks to provide shelter and reduce stress. Tanks were positioned to minimize vibrations from equipment and ensure proper lighting. Staff were trained to avoid sudden movements near housing tanks and to avoid tapping them unless necessary. Breeding: To prevent egg retention and allow recovery, female zebrafish were crossed no more than once every 3 days and at least once every 7–10 days. In tanks with unseparated zebrafish, a sex ratio of approximately one male per two females was maintained, when possible. To minimize aggression and injury during breeding, only adult zebrafish of similar size and age were crossed, and males were never more numerous than females. Handling: The number of animal manipulations and restraint was minimized, and animals were kept or used only until the age of 12 months. Whenever possible and scientifically permissible, animals were reused to maximize data collection. Imaging techniques were employed whenever feasible to gather extensive data from individual animals, reducing the need for repeated procedures. Anesthesia: All surgical procedures and experimental manipulations involving immobilization or embedding were performed under anesthesia with tricaine methanesulfonate (MS-222) to prevent pain or distress, ensuring animal welfare throughout the study. Training and handling: Relevant research staff was trained in zebrafish husbandry practices, including feeding, breeding, egg collection, health monitoring, and rearing to adulthood. Training also covered euthanasia (rapid cooling and freezing), anesthesia, embedding in agarose gel and fin-clipping for genotyping. The animal facility staff primarily managed post-5 dpf rearing.

### Humane endpoints

Humane endpoints were established to prevent or alleviate unnecessary pain or distress. Animal health and behavior were monitored daily, by facility staff during morning feeding and by research staff in the afternoon during the week. Euthanasia was performed when endpoints were met and the condition was progressive, irreversible, or caused persistent suffering, including cases where intervention would not improve the condition or prolonged survival would cause undue distress. When required (excluding age-related criteria), euthanasia was carried out promptly, typically within 24 h on working days and within 48 h on weekends or holidays.

Zebrafish embryos and larvae (≤ 7 dpf) Endpoints: Euthanasia criteria for embryos and larvae included: lack of or severely impaired spontaneous movement, failure to hatch beyond the expected time (>72 hours post-fertilization) with no growth or motility, and lack of response to tactile stimulus. Severe developmental abnormalities, such as pericardial edema, yolk sac edema, spinal curvature (e.g., scoliosis/kyphosis), or an uninflated swim bladder incompatible with survival, also warranted euthanasia. Additionally, reduced or absent heartbeat observed under the microscope, severe pigment loss or abnormal coloration suggestive of systemic failure, and immobility or failure to recover post-anesthesia are criteria for euthanasia.

Zebrafish larvae, juvenile, and adult specimens (> 7 dpf) endpoints: Euthanasia criteria for larvae, juvenile, and adult zebrafish specimens included: prolonged anorexia (more than 2 days), hypoactivity, lethargy, color change, paleness, and a dull appearance. Additional criteria include impaired swimming (erratic, circular movements), abnormal physical appearance, and an inability to maintain position in the water column, with inadequate buoyancy or difficulty staying at a constant level without significant swimming efforts or being in a vertical or inclined position. Loss of balance (incoordination, loss of agility), prolonged social isolation (e.g., the fish moving away from the group when stimulation ceases), and malformations of the fins or spine affecting swimming or behavior are also considered. Other criteria included edema, ascites, protruding scales, abdominal distension, inability to coordinate movement or maintain balance, presence of an abnormal mass, skin lesions, obesity, and aggression causing injury to other fish (such as attacking or biting fins).

### Euthanasia

Euthanasia was performed when humane endpoints were met and the condition was progressive, irreversible, or caused persistent suffering, including cases where intervention was ineffective or prolonged survival would cause unjustifiable distress. Some animals died before meeting euthanasia criteria, mainly larvae (7–20 dpf) during their vulnerable rearing period; these showed no signs of distress the previous day but were found dead the next morning. Occasional juvenile or adult deaths were linked to handling or transfer injuries, and rare cases resulted from suboptimal tank conditions (e.g., ammonia buildup, water flow obstruction). Additionally, some animals were euthanized early to collect biological samples (e.g., RNA, protein) under approved protocols.

The primary euthanasia method was rapid chilling: specimens were immersed for ≥ 40 min in an ice-free container of pre-chilled system water (≤ 4°C) to ensure irreversible hypothermic death. Fish were monitored until loss of equilibrium, cessation of opercular movement, and full immobilization, processed in small batches to avoid piling, and handled only immediately before euthanasia to minimize stress. Secondary euthanasia was usually by freezing after rapid chilling, specimens were transferred with a sieve to labelled disposal bags and frozen at −20°C for ≥ 1 h. For ≤ 3 dpf eggs and embryos, secondary euthanasia consisted of immersion for 1 h in a chilled (2–4°C) bleach solution (1 part sodium hypochlorite 6.15% to 5 parts system water).

### Specimens used, lost and euthanized

Due to changes in the administration of the fish facilities at CRCHUM, records of specimens found dead or euthanized are available only from late 2020 to April 2025. Data for 2019 and most of 2020 could not be retrieved. With the exception of assays that were immediately lethal, experimental fish were euthanized upon completion of procedures in order to minimize unnecessary distress, prevent overcrowding, and avoid discordant growth rates within rearing cohorts intended for subsequent generations. Mortality data for zebrafish specimens older than 30 dpf are presented in Table 4, and specimen usage for dataset generation and the overall project is summarized in Tables 5 and 6, respectively.

**Table 4:** Mortality registry for > 30 dpf zebrafish specimens.

Year	Number of specimens found dead	Number of specimens euthanized
2020	1	43
2021	21	72
2022	100	287
2023	108	305
2024	31	709
2025	3	115

**Table 5:** Number of experimental specimens used to generate the datasets.

Strains	< 5 days post-fertilization	5-20 days post-fertilization	Adults
*C9orf72* ^*+ /+*^	320	2550	4
*C9orf72* ^*-/+*^	200	3147	0
*C9orf72* ^*-/-*^	320	2390	4
*Hb9^-/ +^* *C9orf72* *^+ /+^*	20	0	0
*Hb9^-/ +^* *C9orf72*^*-/-*^	20	0	0
*Hb9* *^+ / +^* *C9orf72* ^*+ /+*^	0	0	0
*Hb9* *^+ / +^* *C9orf72*^*-/-*^	0	0	0

**Table 6:** Number of experimental specimens used for the project.

Strains	< 5 days post-fertilization	5-20 days post-fertilization	Adults
*C9orf72* ^*+ /+*^	1020	4338	20
*C9orf72* ^*-/+*^	260	5099	0
*C9orf72* ^*-/-*^	980	3998	20
*Hb9^-/ +^* *C9orf72* ^*+ /+*^	126	0	0
*Hb9^-/ +^* *C9orf72*^*-/-*^	90	0	0
*Hb9* *^+ / +^* *C9orf72* ^*+ /+*^	114	0	0
*Hb9* *^+ / +^* *C9orf72*^*-/-*^	101	0	0

### sgRNA and Cas9 preparation for C9orf72 knockout line generation

The single-guide RNA (sgRNA) sequence was designed using CRISPRscan to target an early coding region in exon 2 of the C9orf72 gene (ENSDARE00000573949), with the protospacer adjacent motif (PAM) sequence noted in parentheses: GCGCAGCGCAGAGAGCGGCG (CGG). The synthesis of sgRNA and Cas9 mRNA followed the protocols described by Moreno-Mateos et al. (2015) [64]. Microinjections were conducted in Tübingen Long Fin (TL) wild-type zebrafish embryos in accordance with Samarut et al. (2016) [65]. The most likely off-target sites were predicted using CRISPRoff v1.1 [28].

## Genotyping

### Primer design and selection

HRM, PCR and sequencing primers were designed with SnapGene (Dotmatics) version 4.2.1.1 in conjunction with the online tool Primer–BLAST [66]. The second exon of *C9orf72* was sequenced to characterize indels using the following primers: F 5’ GCCAAGACGAAGAACTTGACATCC, R 5’ GGAACAATCTCGGATGACAAC. These primers were designed following the general guidelines established by Chuang et al (2013) [67]. All primer sets are available upon request.

### Fin clip sample collection and DNA extraction

Adult zebrafish were anesthetized in tricaine methanesulfonate (MS-222; Sigma-Aldrich) at 160 mg/L, and a small caudal fin sample was excised using a sharp blade. Fish were immediately transferred back to fresh water in isolated tanks to recover. Genomic DNA extraction was performed in 20 μL of 50 mM NaOH, followed by boiling for 10 minutes. The reaction was then buffered by adding 1/10 volume of 100 mM Tris-HCl (pH 8.0).

### High-resolution melting (HRM)

Identification of the presence of indels in the 2^nd^ exon of *C9orf72* following the injections and genotyping of the specimens from the *C9orf72* KO line was done using the F 5’ CGGAGAGGTCACATTTCTGGCC and R 5’ GCCAAGACGAAGAACTTGACATCC primers. Integration of indels was identifiable by shift in the Δ Fluorescence/ Δ temperature HRM curve profile and the genotyping was done by matching curve profiles of tested specimens to those of specimens whose genotype was confirmed by sequencing.

The PCR reactions were performed as described by Samarut et al. (2016) [65] in a LightCycler 96 (Roche). HRM curves were analyzed using the Roche LightCycler 96 software (version 1.1).

### PCR and sequencing

Second exon sequencing of *C9orf72* for characterization of the indels was done using: F 5’ GCCAAGACGAAGAACTTGACATCC and R: 5’ GGAACAATCTCGGATGACAAC. Sequencing of the 3^rd^ intron of *pou6f1* to validate the absence of off-target indels was done using F 5’ GTCAAATCACCAAACCACCACCCA and R 5’ AGGTGTTCGCAGAAAGCATTGC. Sequencing of the 2^nd^ intron of *si:dkey-277i15.2* to validate the absence of off-target indels was done using F 5’ GCACATGTAGACACTTCGCCTCT and R 5’ ACACATGCTCAATCTTCGTTCTCC. Sequencing of the 11^th^ exon of *ch25hl3* to validate the absence of off-target indels was done using F 5’ GTGCATTGCTTGCTGATGCTAAG, R 5’ CACCGACTGGCACAATGTAGTC.

The PCR reactions were made with 0.5 μL of dNTP (10 μM), 0.5 μL of each primer (10 mM), 2.5 μL of 10x PCR buffer, 0.125 μL of Taq DNA polymerase (GenedireX), 1 μL of genomic DNA and water up to 25 μL. The PCR reaction protocol was 94°C for 5 min, then 35 cycles of 94°C for 30 s, 57–60°C for 30 s and 72°C for 45 s and finally 72°C for 10 min. Samples were sequenced by the Genome Quebec/McGill center using Applied Biosystems 3730xl DNA Analyzer.

## C9orf72 knockout validation

### TaqMan gene expression analysis

To assess *C9orf72* relative transcript expression, we used TaqMan Gene Expression Assays (Applied Biosystems, Thermo Fisher Scientific) with the following probes:

zgc:100846 (C9orf72): Dr03094731_m1

Polr2d (housekeeping gene): Dr03095551_m1

Total RNA was extracted from 30 pooled larvae (2 dpf) using TriReagent® (Sigma-Aldrich) according to the manufacturer’s protocol. RNA was quantified using a NanoPhotometer (Implen) and stored at −80 °C until further use. cDNA was synthesized from 1 µg of total RNA using the Superscript VILO cDNA Synthesis Kit (Thermo Fisher Scientific). Undiluted cDNA was used for real-time PCR, yielding CT values between 25 and 32. Gene expression was quantified using a QuantStudio 3 Real-Time PCR System (Thermo Fisher Scientific) and analyzed using the ΔΔCT method, with Polr2d as the normalization control.

### Western blot

Protein samples for SDS-PAGE and Western blot (WB) analysis were prepared as follows:

Embryo Samples: At 48 hours post-fertilization (hpf), 40 zebrafish embryos per condition were manually dechorionated. Embryos were lysed and homogenized using a pellet pestle in 150 µL of ice-cold lysis buffer containing 150 mM NaCl, 1.0% Triton X-100, 0.1% SDS, 50 mM Tris (pH 7.5), 0.5 mM EDTA, and a protease inhibitor cocktail (1:10, Sigma-Aldrich). Lysates were then boiled for 5 min, centrifuged at 16,000 × g for 10 min at 4°C, and the supernatant was collected. Protein concentration was measured using the Bradford assay (BioRad), and samples were stored at −70°C until further analysis.

Adult Brain Samples: Adult zebrafish (6 months old) were euthanized by prolonged immersion in 300 mg/L MS-222 (Tricaine mesylate; Sigma-Aldrich). Specimens were rinsed with phosphate-buffered saline (PBS) and decapitated using a razor blade. Brains were surgically dissected, pooled (4 brains per condition) into separate 2 mL Eppendorf tubes, weighed, and homogenized on ice with a pellet pestle in 60 µL of ice-cold lysis buffer per 100 mg tissue until no visible tissue remained. Further homogenization was performed by sonication on ice (6 cycles, 20% amplitude, 5 seconds on, 10 seconds off). Subsequent processing steps were identical to those used for embryo samples.

Western Blotting: For Western blot analysis, 85 µg of protein lysate per sample was loaded onto 5–16% gradient polyacrylamide gels and transferred to nitrocellulose membranes. Ponceau S staining was used to visualize transferred proteins. Membranes were blocked in 5% milk in TBST (TBS with 0.1% Tween-20) and incubated overnight at 4°C with the C9orf72 primary antibody (ab221137, 1:2500 dilution in 5% BSA/TBST). The following day, membranes were incubated for 1 hour at room temperature with peroxidase-conjugated secondary antibodies (1:10,000 dilution in 5% milk/TBST), followed by multiple washes. Detection of immunoreactive bands was performed using a LI-COR Odyssey Imaging System (LI-COR Biosciences), and data analysis was conducted with LI-COR Image Studio Lite Version 5.2. Abcam ab221137 epitope is proprietary, correspondence with the manufacturer indicates that the antibody targets the N-terminal half of the protein.

## C9orf72 knockout characterization

### Morphological measurement and gross morphology assessment

Body length and morphological phenotypes were assessed in larvae at 2, 4, 6, 10, 15, and 20 dpf. For each time point, larvae from three independent batches (each containing 20–30 larvae per genotype) were analyzed. For imaging, larvae were individually anesthetized in tricaine methanesulfonate (MS-222; Sigma-Aldrich) at a final concentration of 160 mg/L in E3 medium (5 mM NaCl, 0.17 mM KCl, 0.33 mM CaCl₂, 0.33 mM MgSO₄, 0.00005% w/v methylene blue, pH 7.2). Larvae were then mounted in 0.5% (w/v) low-melting-point agarose (Ultrapure™ LMP Agarose; Invitrogen) in E3 medium on 6 cm petri dishes. Before imaging, dishes were filled with E3 medium to fully submerge the mounted larvae. Lateral images were acquired using a Leica S6E stereomicroscope equipped with an iPhone 6 mounted on an iDu Optics cast from LabCam. Body length was measured from the anterior tip to the caudal peduncle using the FIJI/ImageJ software version 1.53.

### Survival assay

At 24 hpf, 100 embryos per genotype from synchronously initiated crosses, displaying normal growth and no visible defects, were transferred to 1-liter tanks containing 333 mL of E3 medium and maintained in a dry incubator (see Fish Husbandry). Half of the E3 medium was replaced daily, and the volume increased to 500 mL at 10 dpf. Larvae were fed twice daily (see feeding protocols), and dead, moribund, or endpoint-reached specimens (see Humane endpoints) were recorded and removed before each feeding. Every two days, larvae were transferred to clean 1-liter containers to maintain water quality. The assay ended at 20 dpf, unless a sudden, abnormal mortality spike prompted earlier termination.

### Swimming activity monitoring assay

At 6, 8, 10, 12, 15, and 20 dpf, larvae (24 per group for *C9orf72*^*+/+*^ and *C9orf72*^*-/-*^, 48 for *C9orf72*^*-/+*^ mutants) were individually transferred to a 96-well plate containing fresh E3 medium. Swimming activity was recorded under two different paradigms. Phasic paradigm: recording was conducted over a 120-minute alternating dark-light cycle, consisting of six 20-minute phases. Biphasic paradigm: recording was done over an 80-minute alternating dark-light cycle, comprising a 20-minute dark phase followed by a 60-minute light phase. For both paradigms, larvae were placed in a dark recording chamber for 30 minutes to acclimate before recording began. Recordings were performed using a Basler GenIcam camera within a DanioVision recording chamber (Noldus). Ethovision XT 12 (Noldus) was used to quantify total swimming distance, with mean swimming distance per minute and cumulative swimming distance for the entire assay exported for further analysis in GraphPad Prism. Swimming distance was normalized to the mean BL measurement at the corresponding time point for each genotype.

### Spinal primary motoneurons morphology evaluation assay

Transgenic *Hb9:GFP* zebrafish (*Tg(mnx1:GFP)*) were crossed with *C9orf72*^*-/-*^ zebrafish to obtain *Hb9:GFP C9orf72*^*-/+*^ specimens. A stable *Hb9:GFP C9orf72*^*-/-*^ line was established through multiple incrosses. For controls, transgenic *Hb9:GFP* zebrafish were crossed with wild-type TL specimens to generate *Hb9:GFP C9orf72*^*+/+*^, and *Hb9:GFP C9orf72*^*-/+*^ were crossed with *C9orf72*^*-/-*^ to ensure that all GFP-positive embryos were heterozygous for *Hb9:GFP*.

At 30 hours post-fertilization (hpf), embryos expressing GFP in the spinal cord were selected. At 48 hpf, selected embryos were anesthetized using tricaine methanesulfonate (MS-222, Sigma-Aldrich) at a final concentration of 160 mg/L in E3 medium. Once immobilized, embryos were embedded in 0.5% (w/v) low-melting-point agarose (*Ultrapure™ LMP Agarose, Invitrogen*). To visualize spinal primary motor neurons (PMNs) axonal architecture, embryos were imaged using confocal microscopy on an Olympus BX61W1 microscope equipped with a Quorum Technology (Ontario) spinning disk head and a Hamamatsu ORCA-ER camera. All images were acquired using the Volocity (Version 4.1.0, Build 179, Improvision) software and analyzed using the Imaris software (version 8.1.1, Oxford Instruments plc). The first five PMNs caudal to the yolk were imaged at 10 × magnification with an x/y resolution of 1344 × 1024 pixels (pixel size: 0.405 µm) and as z-stacks of 25–30 slices with a z-resolution of 3 µm. Ventral horn distance and the longest ventral projection measurements were obtained with the measurement points and the filament tracing application of Imaris respectively. The first two PMNs units caudal to the yolk were imaged to produce z-stacks (80–90 slices) at 20 × magnification, with an x/y resolution of 512 × 512 pixels (pixel size: 0.203 µm) and a z-resolution of 1 µm. The filament tracing application of Imaris was used to trace the filament of relevant PMNs from which was derived the branching per millimeter of filament and total filament length.

### Neuromuscular junction integrity evaluation assay

At 6 days post-fertilization (dpf), 20 zebrafish larvae per genotype were fixed overnight at 4°C in 4% paraformaldehyde (PFA) in 1 × PBS. The next day, samples were washed three times (3×) for 15 min in 0.1% Tween-20 in PBS (PBS-Tween) at room temperature (RT). To permeabilize tissues, larvae were incubated in 1 mL of 1 mg/mL collagenase (Sigma-Aldrich, C0130-100MG) in PBS for 150 min at RT on a rocker. Afterward, samples were rinsed 3× in 1% Triton X-100 in PBS (PBS-Triton) for 10 min at RT on a rocker. For blocking, larvae were incubated in blocking solution (1% bovine serum albumin, 1% DMSO, 1% Triton X-100, 2% normal goat serum in PBS) for 1 h at RT. To label postsynaptic acetylcholine receptors, larvae were incubated in 1 µg/mL tetramethylrhodamine-conjugated α-bungarotoxin (ThermoFisher, T1175) in 0.1% PBS-Tween for 30 min at RT. After three 15-min washes in PBS-Tween, larvae were incubated overnight at 4°C in blocking solution containing the primary antibody SV2 (1:200, Developmental Studies Hybridoma Bank) to stain presynaptic vesicles. The next day, samples were washed 3× in PBS-Tween for 15 min and incubated overnight at 4°C in 1:1000 Alexa Fluor 488 goat anti-mouse antibody (Jackson ImmunoResearch, 115-545-205) in blocking solution. After a final 3 × PBS-Tween wash (15 min at RT), larvae were stored in 80% glycerol in PBS at 4°C after 1 h at RT on a shaker. For microscopy preparation, larvae were surgically decapitated and mounted in 80% glycerol on 25 × 75 × 1.0 mm microscope slides under a 24 × 50 mm coverslip, with petroleum jelly grease bridges to prevent compression.

The integrity of neuromuscular junctions (NMJs) in spinal motoneurons was analyzed using confocal microscopy on a Zeiss spinning disk Axio Observer Z1 (Carl Zeiss, Germany). For each larva, Z-stacks (115 slices) from the first three spinal hemisegments caudal to the yolk were obtained at 40 × magnification with the following settings: x/y resolution: 512 × 512 pixels (0.33 µm per pixel); z-resolution: 0.44 µm; Tiling: Applied (x or y axis = 2) to capture the full hemisegment; stitched images were employed for analysis. Images were captured using ZEN 2.6.7 Blue edition (Carl Zeiss Microscopy, Germany).

Quantification of NMJ morphology and synaptic alignment was performed using the NMJ Analyser macro implemented in FIJI/ImageJ (version 2.9.0/1.53t) under default settings, as outlined in Singh et al. (2023) [68]. Presynaptic SV2 and postsynaptic α-bungarotoxin (α-BTX) puncta were automatically detected and analyzed for spatial colocalization. Two colocalization thresholds were applied: “colocalizing 100%,” which identifies puncta with complete signal overlap, reflecting high-confidence synaptic contacts; and “colocalizing 50%,” which includes puncta with ≥50% overlap, capturing partially aligned or dynamically remodeling synapses. These complementary thresholds were used to assess synaptic alignment integrity across genotypes.

### Drug validation assay

For all conditions, chemical treatments were administered through in-water dosing, starting at the 64-cell stage (2 hours post-fertilization, hpf) and continued until 6 days post-fertilization (dpf). Vehicle-treated embryos (0.01% dimethyl sulfoxide [DMSO]) served as controls. The compounds tested were pizotifen malate (PM, Cayman Chemical; #20765−500) and melatonin (Cayman Chemical; #14427). Stock solutions were made by dissolving PM (30 mM) and melatonin (10 mM) in 100% DMSO. Working solutions for PM, melatonin, and DMSO vehicle (0.01%) were prepared through sequential dilution in E3 medium and added directly to the wells of a 12-well plate containing 20 zebrafish embryos per well. The medium was refreshed daily to ensure compound stability and minimize degradation. To validate drug effects, swimming activity assays were conducted as previously described. Before the assay, all medium was replaced with fresh E3 medium without DMSO, PM, or melatonin to eliminate any residual effects of acute exposure.

### C. elegans

#### Strains and maintenance.

Standard methods for culturing and handling the worms were employed [69]. Worms were cultured on standard nematode growth medium (NGM) streaked with OP50 *Escherichia coli*. The stock population was maintained at 15°C, while experimental worms were kept at 20°C unless specified otherwise. N2 (wild-type), RB2260 (*alfa-1(ok3062) II)*, EG1285 (*oxIs12 [unc-47p::GFP + lin-15(+)])* and a strain resulting from the cross of RB2260 and EG1285 (*alfa-1(ok3062) II; lin-15B&lin-15A(n765) oxIs12 [unc-47p::GFP + lin-15(+)] X*) were utilized. All experiments were conducted on hermaphrodites. Most strains were supplied by the CGC, which is supported by the NIH Office of Research Infrastructure Programs (P40 OD010440).

#### Paralysis assay.

Day one adult worms were transferred on 5 μM fluorodeoxyuridine (FUDR; Sigma-Aldrich) plates. Worms were scored daily for movement for 12 days. Worms were counted as paralyzed if they failed to move after prodding on the nose. Experiments were performed at 20°C and at least 60 worms were counted per condition.

#### Liquid culture assay.

A synchronized population was obtained using hypochlorite extraction. Worms were grown on solid media until day 1 of adulthood. On day 1, 50 worms per well were placed in S basal with OP50 *E. coli* (optical density 0.5) in a flat-bottom 96-well plate. Measurements were taken using a Microtracker (Phylumtech) with standard parameters for *C. elegans*.

#### Neurodegeneration.

Animals were selected for in vivo visualization on days *9 of* adulthood. They were immobilized in 5 mM levamisole (Sigma-Aldrich) and mounted on 2% agarose pads. Neurons were visualized with a Zeiss Axio Imager M2 microscope. The software used was Zen Pro 2012. At least 30 worms were counted per condition.

### Drug screening

#### Liquid culture.

The animals were prepared for liquid culture as described above. Measurement was performed using a Microtracker (Phylumtech) with standard parameters for *C. elegans*. All compounds were tested at 20 µM.

#### Solid Media.

Worms were exposed from the L4 stage onward to compounds at 20 μM (with 1% DMSO), except for zuclopenthixol, which was used at 2 μM. Compounds were incorporated into NGM solid medium, while worms exposed to NGM medium containing only 1% DMSO served as controls. All plates were streaked with OP50 E. coli. Briefly, 20–40 worms were picked and plated on the corresponding NGM medium (20–40 worms per plate for each condition, and each condition was done in triplicate) to complete observations of paralysis and neurodegeneration.

### Experimental design and statistical analysis

All experiments were conducted in triplicate, and the experimenters were blinded to the specimen genotype whenever possible (e.g., body size and motor neuron measurements). Quantitative data were presented as mean  ±  SEM, box plots, or Kaplan–Meier survival curves. GraphPad Prism v10.3.1 software was used for all statistical analyses. Statistical tests and sample sizes (*n*) are specified in the corresponding figure legends. For all datasets, outliers were eliminated using the ROUT method with a Q = 1%, and analyses were performed on the dataset without the outliers.

## Supporting information

S1 Fig No off-target indels at the three most likely off-target sites for our *C9orf72* KO line. (A) Table listing the predicted off-target sequences, the number of mismatches with the original gRNA, chromosomal coordinates, the overlapping zebrafish gene, and the corresponding genomic region for the three most likely off-target sites, as predicted by CRISPRoff. The uppercase letters indicate matching base pairs and the lowercase letters indicate differing base pairs for the target sequences. (B-D) *C9orf72*^*-/-*^ specimens are genetically identical to *C9orf72*^*+/+*^ controls at the site of the possible off-target cutting predicted in the gene *pou6f1* (B), *si:dkey*-277i15.2 (C), and *ch25hl3* (D) for our gRNA.
(TIFF)

S2 Fig Analysis of eight human/murine C9orf72 antibodies for their ability to detect zebrafish C9orf72. Brain lysates from wild-type (+/+) and C9orf72 KO (-/-) adult zebrafish, as well as whole-larvae lysates at 2 dpf, were prepared and processed for immunoblotting with the indicated C9orf72 antibodies. The red arrows point to positive C9orf72 signals.
(TIFF)

S3 Fig Stereotyped motor activity under alternating light–dark conditions across developmental stages and genotypes in zebrafish larvae. (A-C) Point and connecting line with an error bar graph of the total swimming activity per minute normalized to body length observed with our phasic 120-minute dark-light program for *C9orf72*^*+/+*^, *C9orf72*^*-/+*^ and *C9orf72*^*-/-*^ 10 (A), 15 (B) and 20 (C) days post-fertilization (dpf) larvae. N = 3, n = 72. Data are presented as mean  ±  SEM.
(TIF)

S4 Fig *C9orf72* knockout induces persistent spontaneous swimming activity reduction in zebrafish larvae under single light–dark shift. (A) Typical swimming distance normalized by average body length (BL) per minute pattern observed with our dark (20 min) / light (60 min) program for *C9orf72*^*+/+*^, *C9orf72*^*-/+*^ and *C9orf72*^*-/-*^ 6 days post-fertilization (dpf) larvae. Data are presented as mean  ±  SEM. (B-G) Quantitative analysis of total swimming distance normalized by average BL of larvae at 6, 8, 10, 12, 15 and 20 dpf. (B-D) There is a significant deficit in normalized swimming activity for C9orf72^-/+^ and *C9orf72*^*-/-*^ compared to *C9orf72*^*+/+*^ controls and for *C9orf72*^*-/-*^ compared to *C9orf72*^*-/+*^ specimens at 6, 8 and 10 dpf. (E) *C9orf72*^*-/-*^ and *C9orf72*^*-/+*^ specimens show a significant reduction in normalized swimming compared to *C9orf72*^*+/+*^ controls at 12, 15 and 20 dpf. Statistical tests: Kruskal-Wallis test with Dunn#39;s multiple comparisons post-hoc test (B-F); ordinary one-way ANOVA with Tukey#39;s multiple comparisons post-hoc test (G). **** *p  <  0.0001*, *** *p ≤ 0.001*, ** *p ≤ 0.01*, * *p ≤ 0.05* and NS *p > 0.05*. Boxplot extremities indicate maximum and minimum values, box limits indicate the range of the central 50% of the data, central line marks the median value. N = 3, n = 72 for each genotype except for *C9orf72*^*-/+,*^ where n = 144. N represents the number of experimental repeats from different clutches, and n represents the total number of larvae per genotype considered for the assay.
(TIF)

S5 Fig *C9orf72* knockout induces persistent spontaneous swimming activity reduction in zebrafish larvae under stable light conditions. (A) Typical swimming distance normalized by average body length (BL) per minute pattern observed with our dark (20 min) / light (60 min) program for *C9orf72*^*+/+*^, *C9orf72*^*-/+*^ and *C9orf72*^*-/-*^ 6 days post-fertilization (dpf) larvae. The stable swimming activity period (SSAP) is defined as the period of relatively consistent swimming activity for each genotype, occurring approximately 20 minutes after the dark-to-light transition (minutes 40–80). Data are presented as mean  ±  SEM. (B-G) Quantitative analysis of total swimming distance normalized by average BL of larvae at 6, 8, 10, 12, 15 and 20 dpf during the SSAP period. (B) *C9orf72*^*-/-*^ *and C9orf72*^*-/+*^ specimens show a significant reduction in normalized swimming compared to C9orf72^+/+^ controls at 6 dpf. (C-D) There is a significant deficit in normalized swimming activity for C9orf72^-/+^ and *C9orf72*^*-/-*^ compared to *C9orf72*^*+/+*^ controls and for *C9orf72*^*-/-*^ compared to *C9orf72*^*-/+*^ specimens at 8 and 10 dpf. (E-F) *C9orf72*^*-/-*^ and *C9orf72*^*-/+*^ specimens show a significant reduction in normalized swimming compared to *C9orf72*^*+/+*^ controls at 12, 15. (G) *C9orf72*^*-/-*^ specimens show a significant reduction in normalized swimming compared to *C9orf72*^*+/+*^ at 20dpf. Statistical tests: Kruskal-Wallis test with Dunn#39;s multiple comparisons post-hoc test (B-F), Welch and Brown-Forsythe ANOVA test with Dunnett#39;s T3 multiple comparisons post-hoc test (G). **** *p  <  0.0001*, *** *p ≤ 0.001*, ** *p ≤ 0.01*, * *p ≤ 0.05* and NS *p > 0.05*. Boxplot extremities indicate maximum and minimum value, box limits indicate the range of the central 50% of the data, central line marks the median value. N = 3, n = 72 for each genotype except for *C9orf72*^*-/+,*^ where n = 144. N represents the number of experimental repeats from different clutches, and n represents the total number of larvae per genotype considered for the assay.
(TIF)

S6 Fig Complete *C9orf72* loss induces mild postsynaptic alterations at the neuromuscular junction in zebrafish larvae. (A) Representative confocal images of co-immunostaining at 6 days post-fertilization (dpf) for presynaptic (SV2, green) and postsynaptic (α-bungarotoxin, red) markers in hemisegment NMJs across all three C9orf72-related genotypes. (B) Quantitative analysis reveals a significant increase in the total number of α-bungarotoxin (α-BTX) labeled postsynaptic puncta in *C9orf72*^*-/-*^ larvae compared to *C9orf72*^*-/+*^ and *C9orf72*^*+/+*^ controls. (C) No significant differences were observed in the total number of presynaptic puncta (SV2) across genotypes. (D-E) No significant differences were detected in the number of postsynaptic puncta (α-BTX) that fully colocalize (100%) with presynaptic SV2 or in the number of SV2 puncta fully colocalizing (100%) with α-BTX. (F-G) Similarly, no significant differences were observed in partial colocalization (50%) between α-BTX and SV2 puncta. Statistical tests: Ordinary one-way ANOVA with Tukey#39;s multiple comparisons post-hoc test (B,E,G), Kruskal-Wallis test with Dunn#39;s multiple comparisons post-hoc test (C,F), Welch and Brown-Forsythe ANOVA with Dunnett#39;s T3 multiple comparisons test (D). *** *p ≤ 0.001* and NS *p > 0.05*. Boxplot extremities indicate maximum and minimum values; box limits represent the interquartile range (central 50%), and the central line marks the median value. N = 12 (total number of distinct specimens); n = 34–36 (total number of hemisegments analyzed per genotype). Scale bars = 50 µm.
(TIFF)

S7 Fig Melatonin and pizotifen malate effects on zebrafish larvae spontaneous swimming activity across genotypes and concentrations. Melatonin treatment did not improve the swimming deficit in *C9orf72* KO larvae at any tested concentration, nor did higher concentrations of PM. Bar graphs represent the total swimming distance of 6 dpf zebrafish subjected to the phasic light-dark paradigm. (A-C) Melatonin treatment had no significant effect on total swimming activity across all genotypes and concentrations tested excepted for a reduction of swimming activity *C9orf72*^*-/+*^ at 1 µm. (D-F) Exposure to the highest doses of PM significantly reduced swimming activity in *C9orf72*^*+/+*^ and *C9orf72*^*-/+*^ larvae, suggesting potential toxicity (D), whereas no significant effect was observed for any genotype at other tested doses (E-F). Statistical tests: Kruskal-Wallis test with Dunn#39;s multiple comparisons post-hoc test (B-F), Welch and Brown-Forsythe ANOVA with Dunnett#39;s T3 multiple comparisons test (A). **** *p  <  0.0001*, ** *p ≤ 0.01*, NS *p > 0.05*. (-) indicates untreated specimens, (+) indicates treated specimens. Sample size: N = 2–4 (number of independent swimming assays from different clutches); n = 32–80 (total number of individual specimens). Data are presented as mean  ±  SEM.
(TIFF)

S1 Table Body length averages and relevant statistics for zebrafish larvae of all three *C9orf72* knockout genotypes. Table of all the results and associated statistics concerning the measurement of body length (BL) in millimeters (mm) of *C9orf72*^*+/+*^, *C9orf72*^*-/*+^, and *C9orf72*^*-/-*^ specimens for all the time points considered. Days post-fertilization (dpf); standard error of the mean (SEM).
(CSV)

S2 Table Oligonucleotide sequences. Table of all the oligonucleotide sequences that were used for the generation of the *C9orf72* LOF line by CRISPR/Cas9, PCR amplification, HRM, and sequencing.
(XLSX)

S3 Table Antibodies. Table of all the antibodies used for the immunofluorescence assay (neuromuscular junction imaging) and the Western blots (C9orf72 detection).
(XLSX)

S4 Table Molecules identified that ameliorate *alfa-1(ok3062)* motility defect in liquid culture. Table of the 80 compounds identified that were able to ameliorate the motility defect of *alfa-1(ok3062) C*. elegans animals in liquid culture.
(XLSX)

S5 Table Molecules identified that improve *alfa-1(ok3062)* age-dependent paralysis and neurodegeneration phenotypes. Table of the molecules identified that improve the age-dependent paralysis and neurodegeneration phenotypes of *alfa-1(ok3062) C. elegans* animals on solid media. Statistical tests and parameters are the same as described for melatonin and PM in supporting data.
(XLSX)
