# Supplementary figures and images for: Characterization of a C9orf72 Knockout Danio rerio model for ALS and cross-species validation of potential therapeutics screened in Caenorhabditis elegans
Source: PLoS One. 2026 Apr 10;21(4):e0346613. doi: 10.1371/journal.pone.0346613 (PMC13068224; doi:10.1371/journal.pone.0346613)

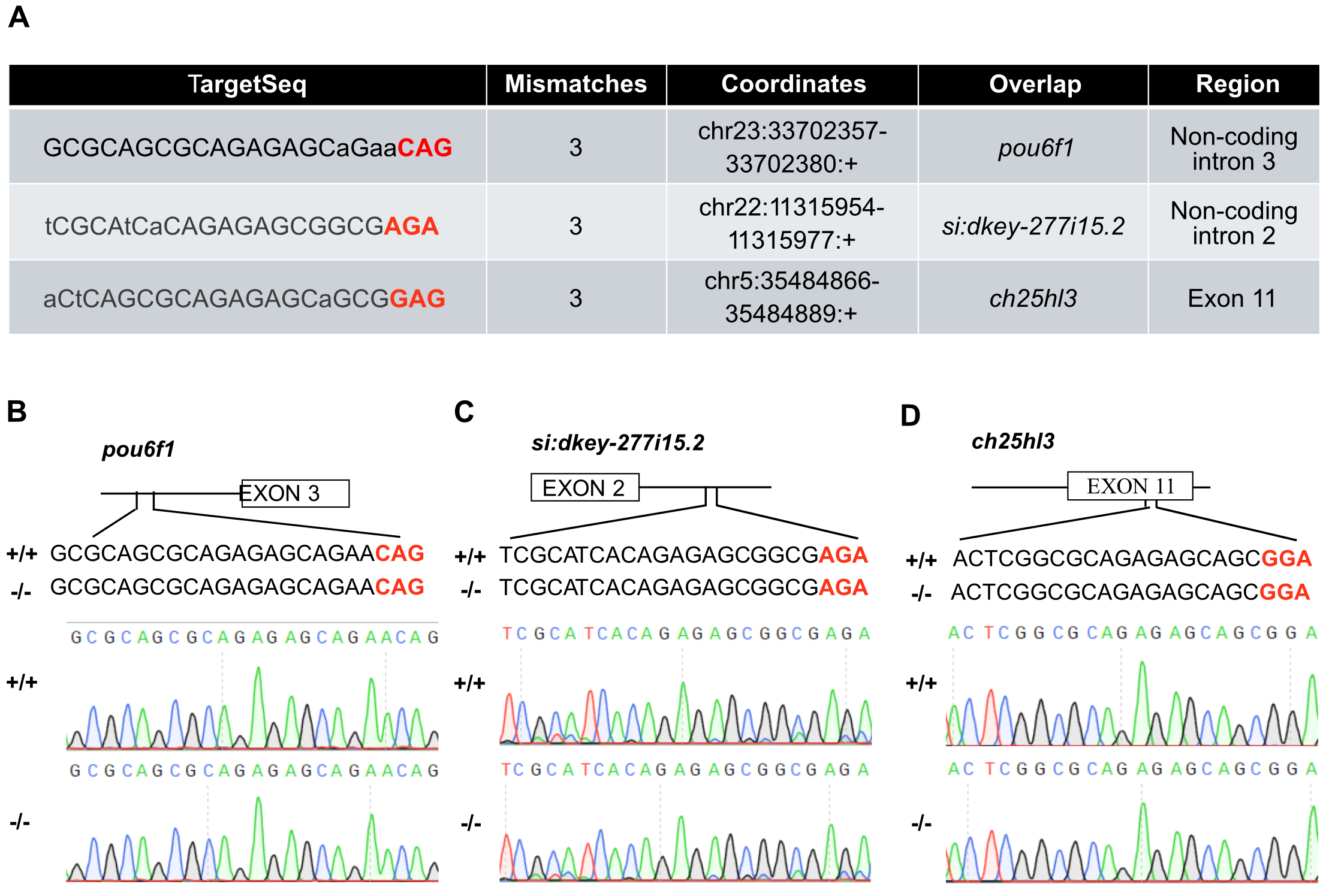

Supplement: S1 Fig — (A) Table listing the predicted off-target sequences, the number of mismatches with the original gRNA, chromosomal coordinates, the overlapping zebrafish gene, and the corresponding genomic region for the three most likely off-target sites, as predicted by CRISPRoff. The uppercase letters indicate matching base pairs and the lowercase letters indicate differing base pairs for the target sequences. (B-D) C9orf72-/- specimens are genetically identical to C9orf72+/+ controls at the site of the possible off-target cutting predicted in the gene pou6f1 (B), si:dkey-277i15.2 (C), and ch25hl3 (D) for our gRNA. (TIFF) [file pone.0346613.s001.tiff]

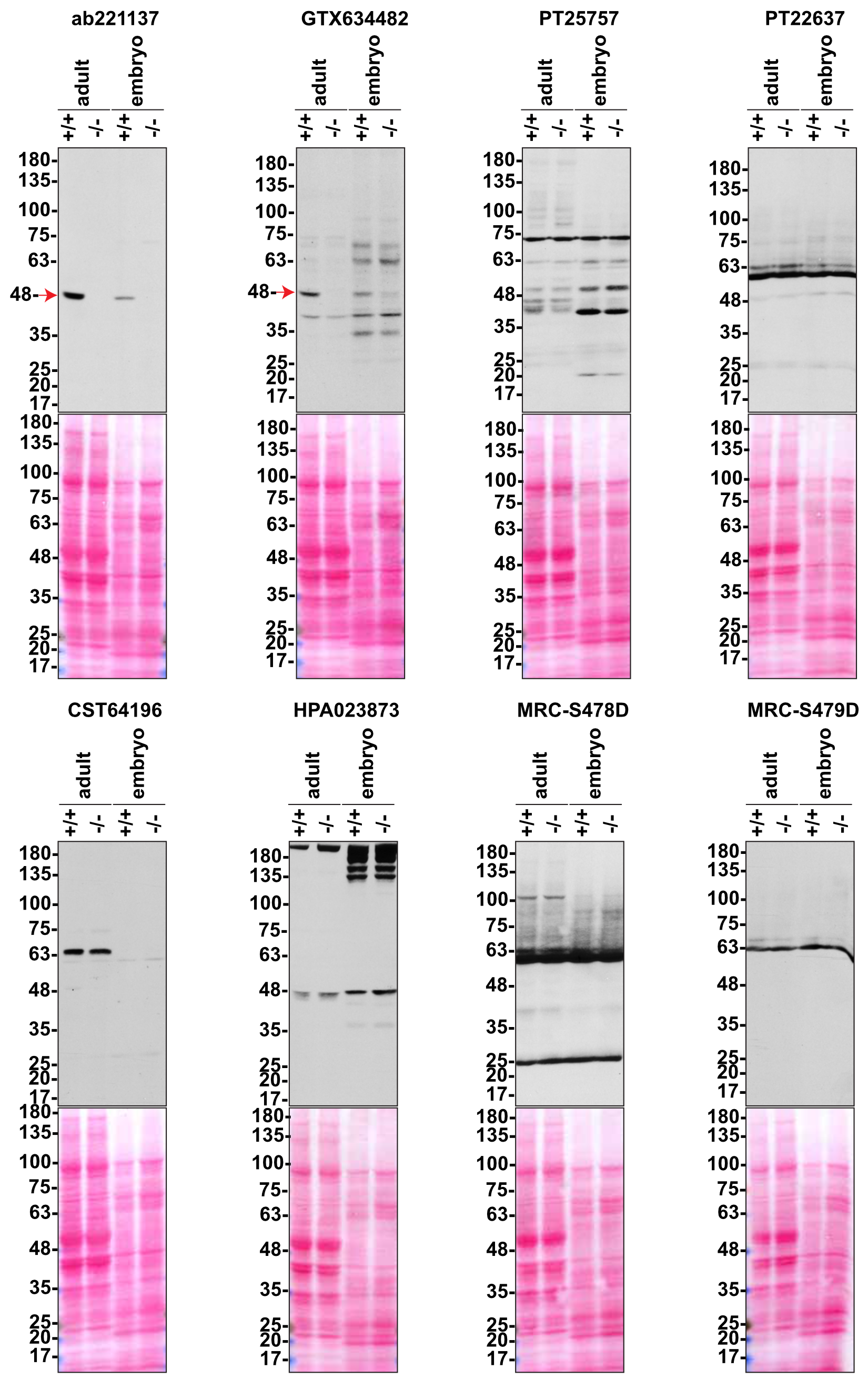

Supplement: S2 Fig — Brain lysates from wild-type (+/+) and C9orf72 KO (-/-) adult zebrafish, as well as whole-larvae lysates at 2 dpf, were prepared and processed for immunoblotting with the indicated C9orf72 antibodies. The red arrows point to positive C9orf72 signals. (TIFF) [file pone.0346613.s002.tiff]

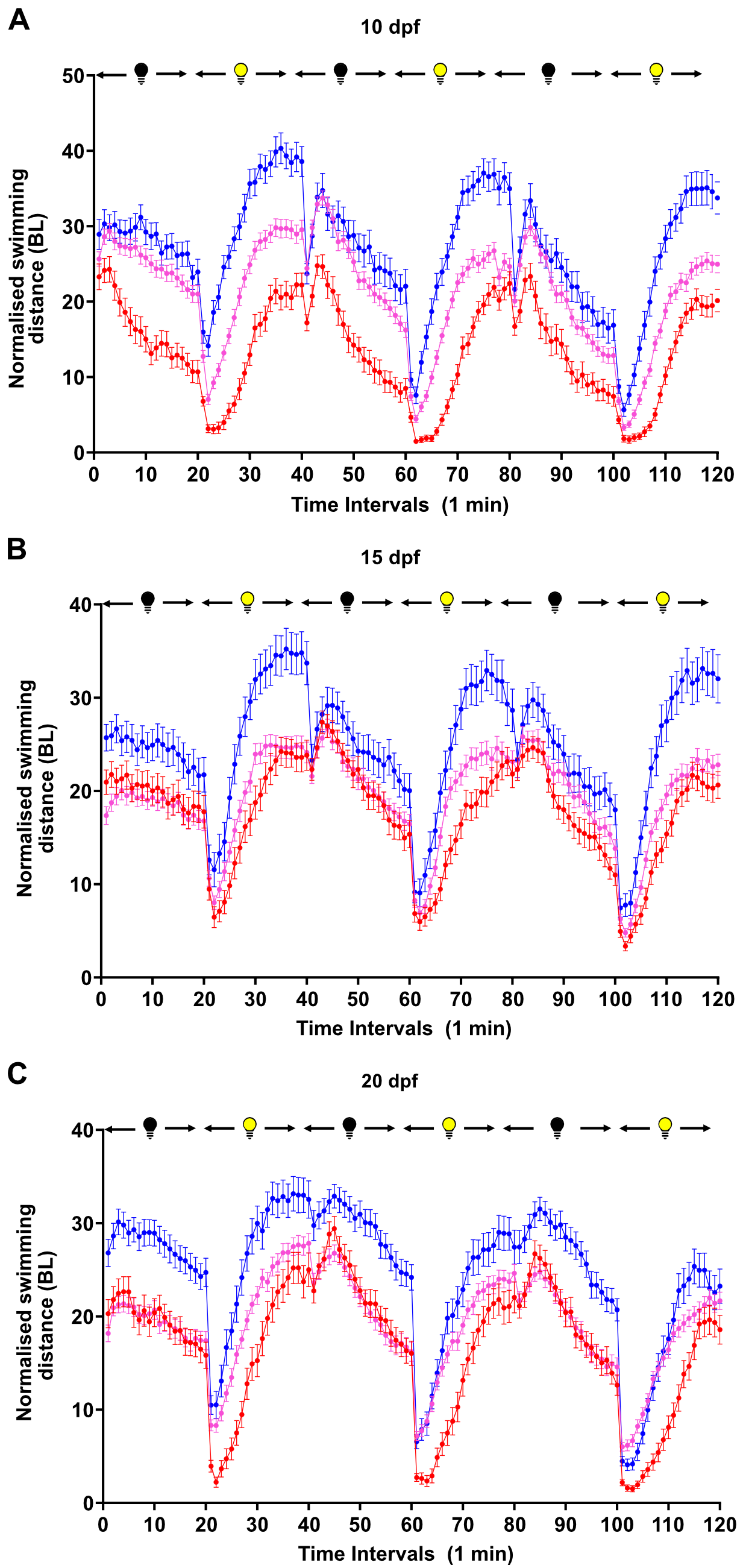

Supplement: S3 Fig — (A-C) Point and connecting line with an error bar graph of the total swimming activity per minute normalized to body length observed with our phasic 120-minute dark-light program for C9orf72+/+, C9orf72-/+ and C9orf72-/- 10 (A), 15 (B) and 20 (C) days post-fertilization (dpf) larvae. N = 3, n = 72. Data are presented as mean ± SEM. (TIF) [file pone.0346613.s003.tif]

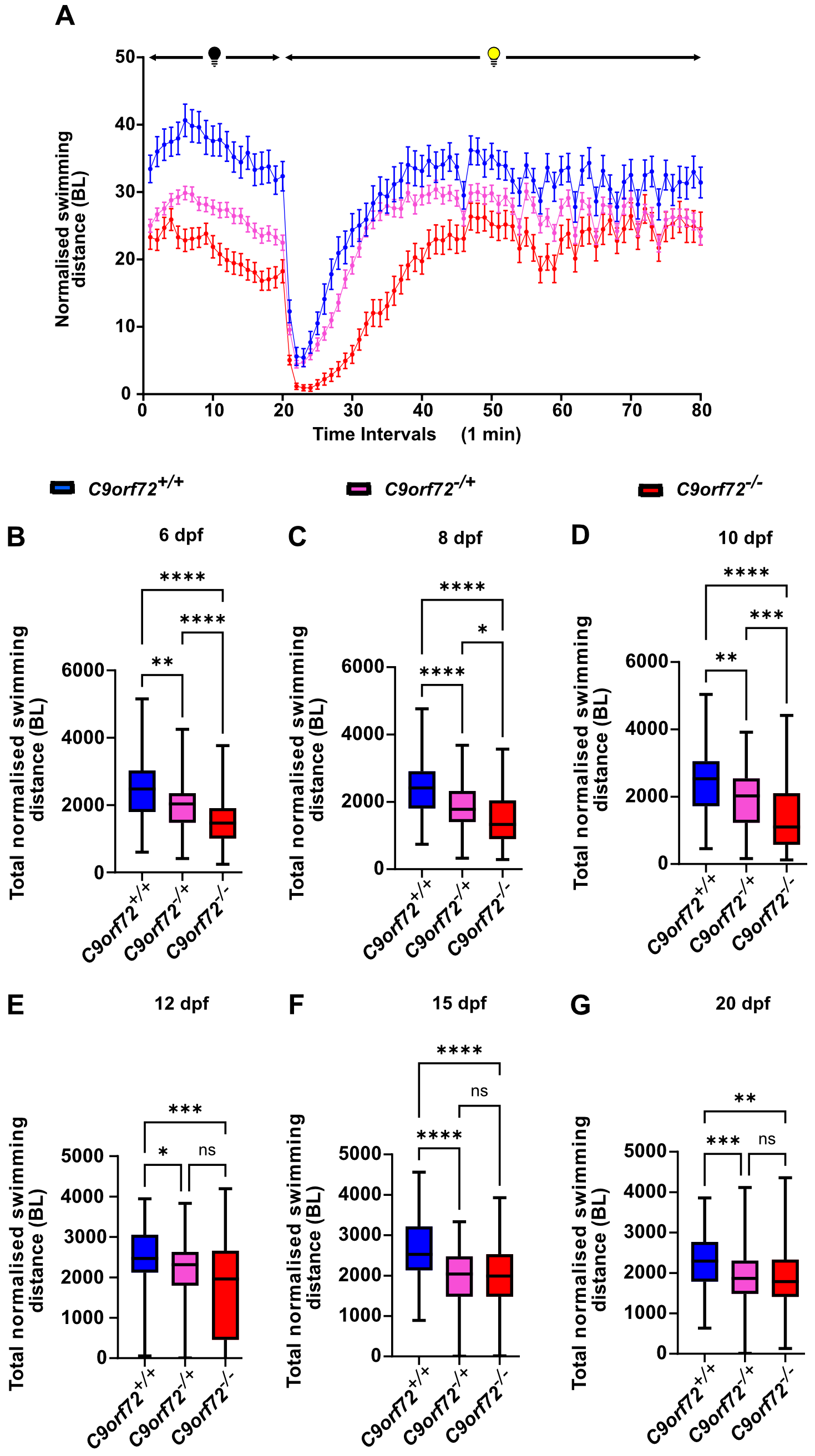

Supplement: S4 Fig — (A) Typical swimming distance normalized by average body length (BL) per minute pattern observed with our dark (20 min) / light (60 min) program for C9orf72+/+, C9orf72-/+ and C9orf72-/- 6 days post-fertilization (dpf) larvae. Data are presented as mean ± SEM. (B-G) Quantitative analysis of total swimming distance normalized by average BL of larvae at 6, 8, 10, 12, 15 and 20 dpf. (B-D) There is a significant deficit in normalized swimming activity for C9orf72-/+ and C9orf72-/- compared to C9orf72+/+ controls and for C9orf72-/- compared to C9orf72-/+ specimens at 6, 8 and 10 dpf. (E) C9orf72-/- and C9orf72-/+ specimens show a significant reduction in normalized swimming compared to C9orf72+/+ controls at 12, 15 and 20 dpf. Statistical tests: Kruskal-Wallis test with Dunn#39;s multiple comparisons post-hoc test (B-F); ordinary one-way ANOVA with Tukey#39;s multiple comparisons post-hoc test (G). **** p < 0.0001, *** p ≤ 0.001, ** p ≤ 0.01, * p ≤ 0.05 and NS p > 0.05. Boxplot extremities indicate maximum and minimum values, box limits indicate the range of the central 50% of the data, central line marks the median value. N = 3, n = 72 for each genotype except for C9orf72-/+, where n = 144. N represents the number of experimental repeats from different clutches, and n represents the total number of larvae per genotype considered for the assay. (TIF) [file pone.0346613.s004.tif]

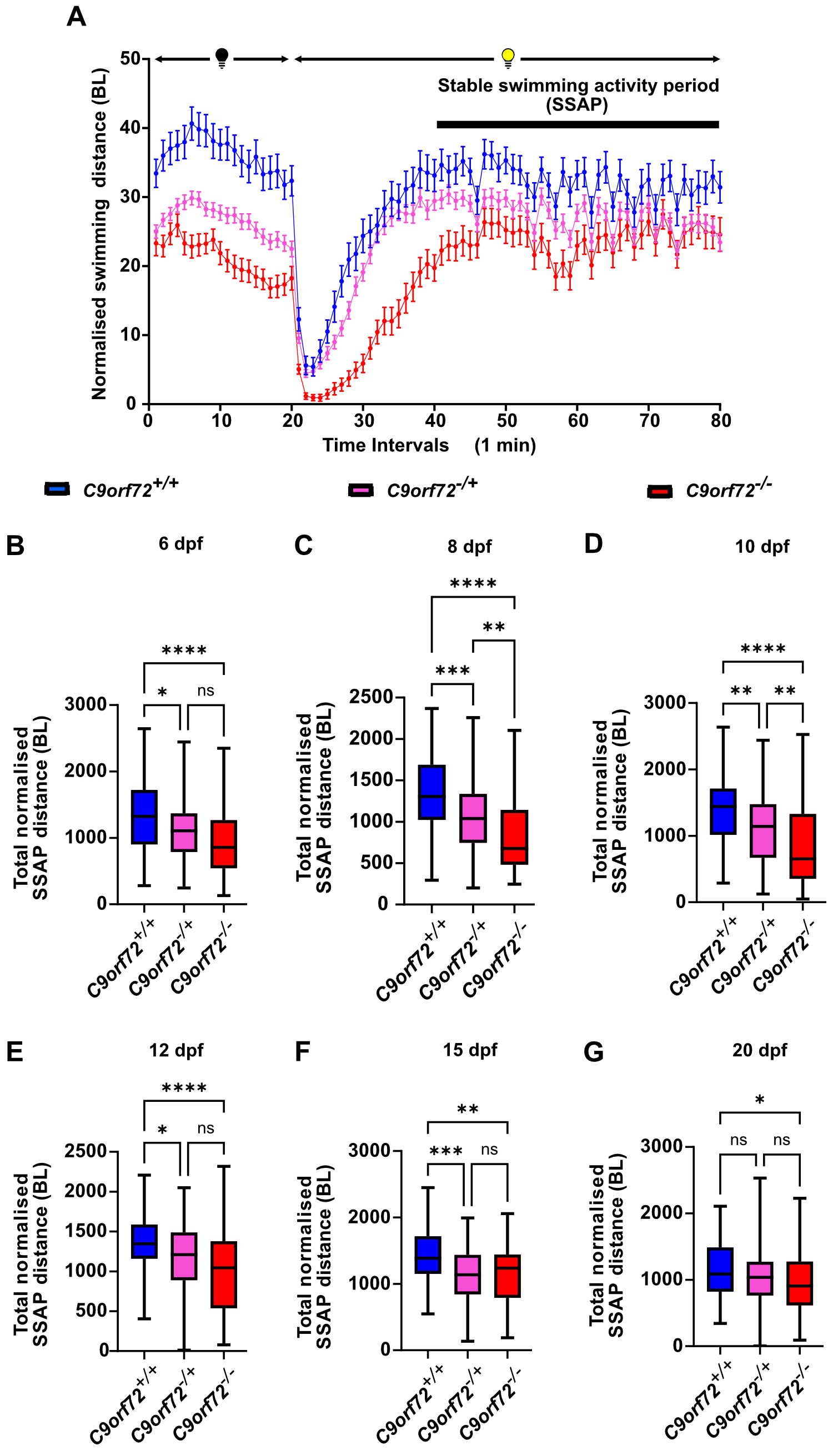

Supplement: S5 Fig — (A) Typical swimming distance normalized by average body length (BL) per minute pattern observed with our dark (20 min) / light (60 min) program for C9orf72+/+, C9orf72-/+ and C9orf72-/- 6 days post-fertilization (dpf) larvae. The stable swimming activity period (SSAP) is defined as the period of relatively consistent swimming activity for each genotype, occurring approximately 20 minutes after the dark-to-light transition (minutes 40–80). Data are presented as mean ± SEM. (B-G) Quantitative analysis of total swimming distance normalized by average BL of larvae at 6, 8, 10, 12, 15 and 20 dpf during the SSAP period. (B) C9orf72-/- and C9orf72-/+ specimens show a significant reduction in normalized swimming compared to C9orf72+/+ controls at 6 dpf. (C-D) There is a significant deficit in normalized swimming activity for C9orf72-/+ and C9orf72-/- compared to C9orf72+/+ controls and for C9orf72-/- compared to C9orf72-/+ specimens at 8 and 10 dpf. (E-F) C9orf72-/- and C9orf72-/+ specimens show a significant reduction in normalized swimming compared to C9orf72+/+ controls at 12, 15. (G) C9orf72-/- specimens show a significant reduction in normalized swimming compared to C9orf72+/+ at 20dpf. Statistical tests: Kruskal-Wallis test with Dunn#39;s multiple comparisons post-hoc test (B-F), Welch and Brown-Forsythe ANOVA test with Dunnett#39;s T3 multiple comparisons post-hoc test (G). **** p < 0.0001, *** p ≤ 0.001, ** p ≤ 0.01, * p ≤ 0.05 and NS p > 0.05. Boxplot extremities indicate maximum and minimum value, box limits indicate the range of the central 50% of the data, central line marks the median value. N = 3, n = 72 for each genotype except for C9orf72-/+, where n = 144. N represents the number of experimental repeats from different clutches, and n represents the total number of larvae per genotype considered for the assay. (TIF) [file pone.0346613.s005.tif]

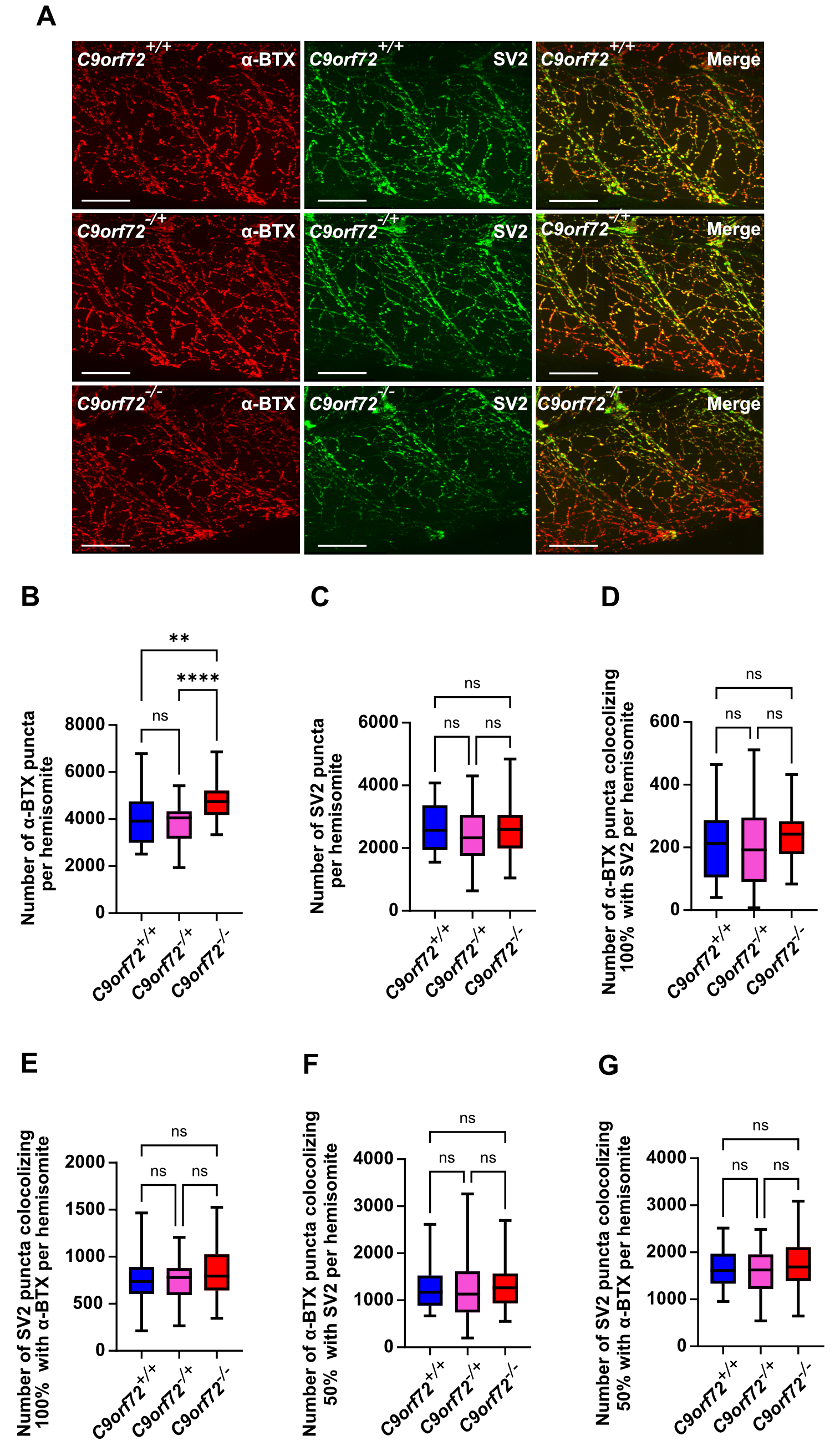

Supplement: S6 Fig — (A) Representative confocal images of co-immunostaining at 6 days post-fertilization (dpf) for presynaptic (SV2, green) and postsynaptic (α-bungarotoxin, red) markers in hemisegment NMJs across all three C9orf72-related genotypes. (B) Quantitative analysis reveals a significant increase in the total number of α-bungarotoxin (α-BTX) labeled postsynaptic puncta in C9orf72-/- larvae compared to C9orf72-/+ and C9orf72+/+ controls. (C) No significant differences were observed in the total number of presynaptic puncta (SV2) across genotypes. (D-E) No significant differences were detected in the number of postsynaptic puncta (α-BTX) that fully colocalize (100%) with presynaptic SV2 or in the number of SV2 puncta fully colocalizing (100%) with α-BTX. (F-G) Similarly, no significant differences were observed in partial colocalization (50%) between α-BTX and SV2 puncta. Statistical tests: Ordinary one-way ANOVA with Tukey#39;s multiple comparisons post-hoc test (B,E,G), Kruskal-Wallis test with Dunn#39;s multiple comparisons post-hoc test (C,F), Welch and Brown-Forsythe ANOVA with Dunnett#39;s T3 multiple comparisons test (D). *** p ≤ 0.001 and NS p > 0.05. Boxplot extremities indicate maximum and minimum values; box limits represent the interquartile range (central 50%), and the central line marks the median value. N = 12 (total number of distinct specimens); n = 34–36 (total number of hemisegments analyzed per genotype). Scale bars = 50 µm. (TIFF) [file pone.0346613.s006.tiff]

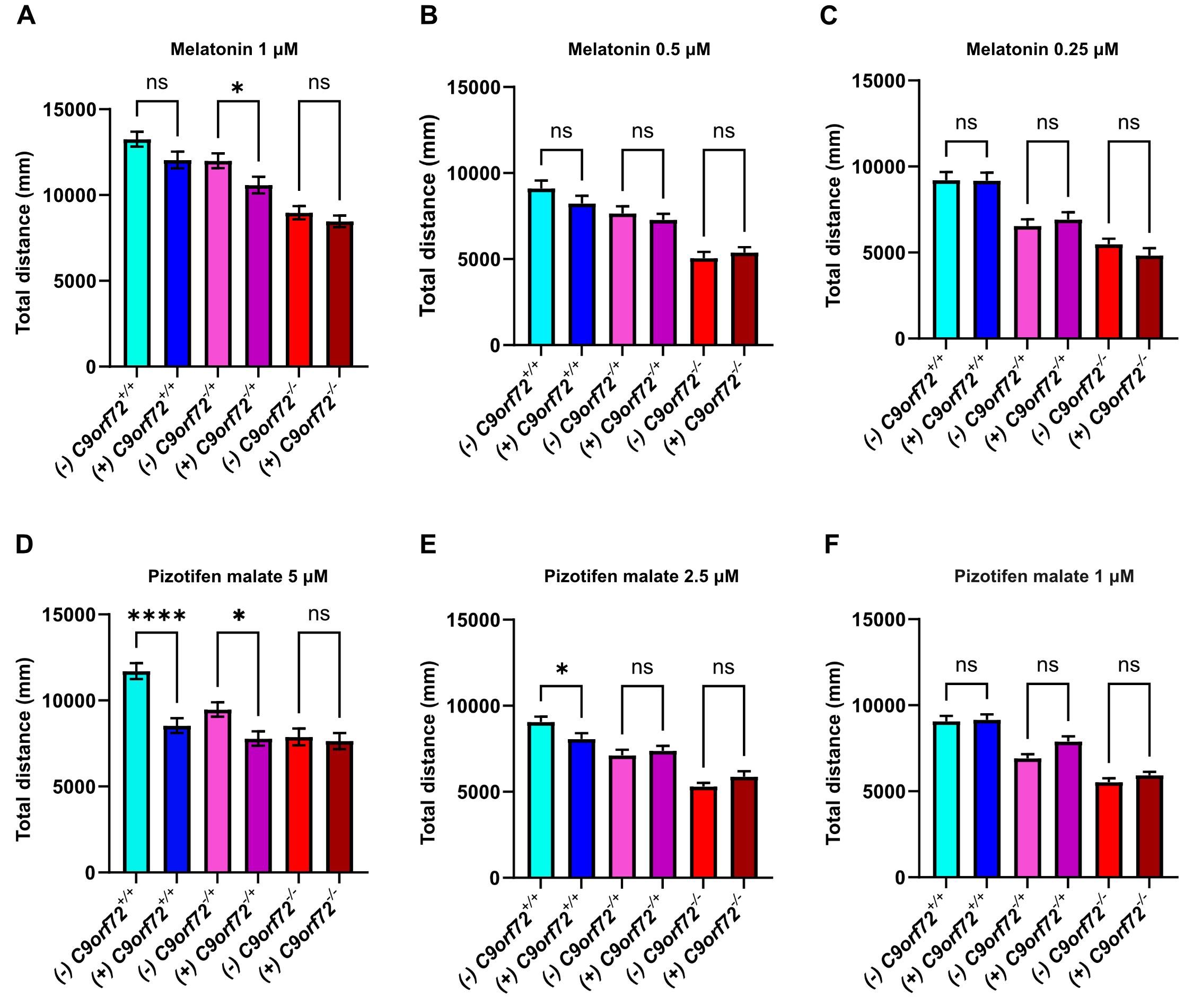

Supplement: S7 Fig — Melatonin treatment did not improve the swimming deficit in C9orf72 KO larvae at any tested concentration, nor did higher concentrations of PM. Bar graphs represent the total swimming distance of 6 dpf zebrafish subjected to the phasic light-dark paradigm. (A-C) Melatonin treatment had no significant effect on total swimming activity across all genotypes and concentrations tested excepted for a reduction of swimming activity C9orf72-/+ at 1 µm. (D-F) Exposure to the highest doses of PM significantly reduced swimming activity in C9orf72+/+ and C9orf72-/+ larvae, suggesting potential toxicity (D), whereas no significant effect was observed for any genotype at other tested doses (E-F). Statistical tests: Kruskal-Wallis test with Dunn#39;s multiple comparisons post-hoc test (B-F), Welch and Brown-Forsythe ANOVA with Dunnett#39;s T3 multiple comparisons test (A). **** p < 0.0001, ** p ≤ 0.01, NS p > 0.05. (-) indicates untreated specimens, (+) indicates treated specimens. Sample size: N = 2–4 (number of independent swimming assays from different clutches); n = 32–80 (total number of individual specimens). Data are presented as mean ± SEM. (TIFF) [file pone.0346613.s007.tiff]
